# Hippocampal ceRNA networks from chronic intermittent ethanol vapor-exposed male mice and functional analysis of top-ranked lncRNA genes for ethanol drinking phenotypes

**DOI:** 10.3389/adar.2022.10831

**Published:** 2022-12-05

**Authors:** Sonja L. Plasil, Valerie J. Collins, Annalisa M. Baratta, Sean P. Farris, Gregg E. Homanics

**Affiliations:** ^1^ Department of Pharmacology and Chemical Biology, School of Medicine, University of Pittsburgh, Pittsburgh, PA, United States; ^2^ Center for Neuroscience, School of Medicine, University of Pittsburgh, Pittsburgh, PA, United States; ^3^ Department of Anesthesiology and Perioperative Medicine, School of Medicine, University of Pittsburgh, Pittsburgh, PA, United States; ^4^ Department of Biomedical Informatics, School of Medicine, University of Pittsburgh, Pittsburgh, PA, United States; ^5^ Department of Neurobiology, School of Medicine, University of Pittsburgh, Pittsburgh, PA, United States

**Keywords:** alcohol use disorder, ethanol consumption, CRISPR/Cas9, long-noncoding RNA, transcriptome, knockout, mutagenesis, epigenetics

## Abstract

The molecular mechanisms regulating the development and progression of alcohol use disorder (AUD) are largely unknown. While noncoding RNAs have previously been implicated as playing key roles in AUD, long-noncoding RNA (lncRNA) remains understudied in relation to AUD. In this study, we first identified ethanol-responsive lncRNAs in the mouse hippocampus that are transcriptional network hub genes. Microarray analysis of lncRNA, miRNA, circular RNA, and protein coding gene expression in the hippocampus from chronic intermittent ethanol vapor- or air- (control) exposed mice was used to identify ethanol-responsive competing endogenous RNA (ceRNA) networks. Highly interconnected lncRNAs (genes that had the strongest overall correlation to all other dysregulated genes identified) were ranked. The top four lncRNAs were novel, previously uncharacterized genes named *Gm42575*, *4930413E15Rik*, *Gm15767*, and *Gm33447*, hereafter referred to as Pitt1, Pitt2, Pitt3, and Pitt4, respectively. We subsequently tested the hypothesis that CRISPR/Cas9 mutagenesis of the putative promoter and first exon of these lncRNAs in C57BL/6J mice would alter ethanol drinking behavior. The Drinking in the Dark (DID) assay was used to examine binge-like drinking behavior, and the Every-Other-Day Two-Bottle Choice (EOD-2BC) assay was used to examine intermittent ethanol consumption and preference. No significant differences between control and mutant mice were observed in the DID assay. Female-specific reductions in ethanol consumption were observed in the EOD-2BC assay for Pitt1, Pitt3, and Pitt4 mutant mice compared to controls. Male-specific alterations in ethanol preference were observed for Pitt1 and Pitt2. Female-specific increases in ethanol preference were observed for Pitt3 and Pitt4. Total fluid consumption was reduced in Pitt1 and Pitt2 mutants at 15% v/v ethanol and in Pitt3 and Pitt4 at 20% v/v ethanol in females only. We conclude that all lncRNAs targeted altered ethanol drinking behavior, and that lncRNAs Pitt1, Pitt3, and Pitt4 influenced ethanol consumption in a sex-specific manner. Further research is necessary to elucidate the biological mechanisms for these effects. These findings add to the literature implicating noncoding RNAs in AUD and suggest lncRNAs also play an important regulatory role in the disease.

## Introduction

Alcohol use disorder (AUD) is a chronic and debilitating neurological disorder that has extensive global, social, and economic burdens. In the United States AUD is one of the leading risk factors for premature death and disability (1) and has an annual estimated socioeconomic cost of ∼$250 billion (2). Many consequences of chronic alcohol misuse are attributed to alcohol’s effect on the brain (3, 4), and alcohol acts in part by altering neural gene expression (4–8). Deciphering alcohol’s impact on gene expression within discrete brain regions and subsequent downstream effects offers an opportunity to identify novel pharmacological targets that could prevent sustained alcohol-induced alterations from occurring in humans.

The hippocampus is an important ethanol-sensitive brain region involved in the transition to AUD (9–11). The hippocampus is susceptible to the detrimental impacts of excessive alcohol exposure (12–14), and binge-like ethanol consumption has been shown to significantly impact neuroimmune functions within the hippocampus in mice (15). Neuroimmune, transcriptional, and epigenetic cell signaling changes are shown to underly the loss of hippocampal neurogenesis (15, 17–20) and plasticity (9, 19, 21) following both exposure to ethanol and other drugs of abuse (17, 19, 22, 23). This supports the concept that hippocampal neuroadaptations are critical targets to understand ethanol withdrawal and consumption.

The noncoding RNA (ncRNA) transcriptome acts as epigenetic regulators controlling cellular homeostasis (24). Evidence supports important roles for ncRNA in the progression of AUD (7, 8, 25–27). Functional studies targeting specific RNAs in animal models for AUD have shown that the ethanol-responsive RNA transcriptome is involved in ethanol consumption, withdrawal, and the progression of addiction. Transcriptome data gathered from both humans and animals chronically exposed to ethanol has revealed mass dysregulation of multiple RNA subtypes in the brain (7, 8), such as mRNAs and their coded proteins (28–34), miRNAs (7, 35–39), circular RNAs (circRNA) (40), and long noncoding RNAs (lncRNAs) (4, 41–43). LncRNAs are an abundant and diverse subclass of ncRNAs defined as transcripts exceeding 200 nucleotides (nts) that do not encode protein (7, 44). There are over 100,000 different lncRNA transcripts (45–49), with many showing brain-specific expression (50). LncRNAs are known for their roles in epigenetic regulation (44, 50–53), such as impacting chromatin modifications, RNA processing events, modulation of miRNAs, gene silencing, regulation of neighboring genes, synaptic plasticity (44) and molecular networks by acting and interacting as central hubs (8, 54). Those that have been studied largely function by regulating gene expression through *cis*- and *trans*-mechanisms (55, 56). LncRNA expression can be developmentally regulated, can show tissue- and cell-type specific expression, and can be involved in numerous cellular pathways critical to normal development and physiology (50–53, 57–59). The dysregulation of lncRNAs has been linked to the pathophysiology of several disease states (7, 8, 41, 44, 53, 60–66) including AUD (41, 67, 68), drug addiction (63, 69–71), psychiatric disorders (72, 73), and stress responses (74, 75). Identifying and directly testing lncRNAs that regulate ethanol consumption and related behaviors is important to fully understand the initiation and progression of AUD. Here, we hypothesize that specific ethanol-responsive lncRNAs are critical hubs of molecular networks that act as key determinants of ethanol consumption. Targeting specific ethanol-responsive lncRNAs for genetic modulation that have strong correlations to other ethanol-responsive RNAs may help discern transcriptomic network alterations that can impact ethanol drinking phenotypes.

To shed light on how ncRNAs interact with each other *in vivo*, competing endogenous RNA (ceRNA) networks can be bioinformatically generated from transcriptome data sets (76–81). LncRNA, circRNA, and miRNA are all known as ncRNA epigenetic regulators, which work in concert to coordinate mRNA expression, protein levels, and homeostasis *via* such functions as transcription factors, molecular sponges, scaffolds, decoys, and guides (for reviews, see: (7,24, 44, 51, 53, 54, 63). These networks provide insight into discrete clusters of RNAs that interact and/or compete with each other to maintain the network’s function (76–81). These correlated RNAs can then be intertwined and linked together computationally to either increase or decrease the rank of hub genes based on their relative interconnectivity with other genes. Generating ethanol-responsive ceRNA networks from four prominent RNA subtypes, lncRNA, mRNA, circRNA, and miRNA, allowed for novel networks and hub genes to be identified in the present study. A list of top-ranked putative hub ethanol-responsive lncRNAs was generated and genes were prioritized for functional interrogation *via* CRISPR/Cas9 mutagenesis.

The acquisition of transcriptome data has greatly outpaced our capacity to functionally study genes *in vivo* that are hypothesized to contribute to AUD (82). To circumvent this bottleneck, we recently developed an accelerated CRISPR/Cas9 approach to create a cohort of functional KnockOut (KO) animals in a single generation (83). Here we applied this CRISPR Turbo Accelerated KO (CRISPy TAKO) methodology to test the hypothesis that mutation of ethanol-responsive lncRNAs identified from hippocampal ceRNA network analyses impact ethanol drinking behavior. We tested the top four lncRNAs that were identified as potential hubs for ethanol-responsive networks *via* ceRNA analysis. We generated four CRISPy TAKO mouse lines targeting the top four lncRNA candidates identified: *Gm42575*, *4930413E15Rik*, *Gm15767*, and *Gm33447*, hereafter referred to as Pitt1, Pitt2, Pitt3, and Pitt4, respectively. All gene-targeted cohorts were tested for binge-like drinking behavior and intermittent ethanol consumption and preference.

## Materials and methods

### Animals

All experiments were approved by the Institutional Animal Care and Use Committee of the University of Pittsburgh and conducted in accordance with the National Institutes of Health Guidelines for the Care and Use of Laboratory Animals. C57BL/6J male and female mice used for chronic intermittent ethanol vapor (CIEV) exposure, generation of embryos for electroporation, and purchased control groups were procured from The Jackson Laboratory (Bar Harbor, ME). CD-1 recipient females and vasectomized males were procured from Charles River Laboratories, Inc. (Wilmington, MA). Mice were housed in individually ventilated caging under specific pathogen-free conditions with 12-h light/dark cycles (lights on at 7 AM) and had *ad libitum* access to food (irradiated 5P76 ProLab IsoProRMH3000; LabDiet, St. Louis, MO) and water.

### Chronic intermittent ethanol vapor exposure

Male mice were exposed to a 16-h CIEV or room-air paradigm as previously reported (84) (*n* = 5–6/treatment). Briefly, mice were given a priming intraperitoneal injection of either 1.5 g/kg ethanol (Decon Labs, Inc., #2716GEA) and 68 mg/kg pyrazole (Sigma-Aldrich, P56607-5G) or saline and 68 mg/kg pyrazole, then immediately subjected to vaporized ethanol or room air (respectively) for 16 h/day, 4 days/week, for 7 weeks. Hippocampal tissue was harvested 24 h following the final vapor exposure.

### Total RNA isolation and microarray profiling

Left hippocampi were homogenized in 1 ml TRIzol reagent (Invitrogen, #15596018) and sent to Arraystar Inc. (Rockville, MD) for transcriptome analysis. For circRNA analysis, Arraystar Inc. isolated total RNA, digested with RNase R (Epicentre, Inc.), fluorescently labeled (Arraystar Super RNA Labeling Kit), and subsequently hybridized to Arraystar Mouse circRNA Array V2 (8 × 15K). For lncRNA and mRNA analysis, Arraystar Inc. isolated rRNA depleted RNA (mRNA-ONLY™ Eukaryotic mRNA Isolation Kit, Epicentre) from total RNA. rRNA depleted RNA was amplified, fluorescently labeled (Arraystar Flash RNA Labeling Kit), and hybridized to Agilent Arrays (Mouse LncRNA Array v3.0, 8 × 60K). An Agilent Scanner G2505C was used to scan the arrays. The University of Pittsburgh Genomics Sequencing Core used Applied Biosystems GeneChip miRNA 4.0 Arrays to measure changes in abundance of miRNAs from the total RNA samples isolated from the hippocampal tissue. The median intensity expression values were log_2_ transformed and quantile normalized across samples. Differential expression were determined using linear models for microarray data (limma) (85) with nominal *p*-value less than or equal to 0.05 as statistically significant. Weighted gene co-expression network (WGCNA) was used to determine all pairwise correlation among RNAs (i.e., lncRNA, mRNA, circRNA, miRNA) across samples. An unsigned network was constructed using minimum module size of 100, a cut height of 0.99, and a power of 6 to approximate a scale-free topology. The expression of unassigned RNAs were labeled as gray. The total connectivity of individual probes was determined from the pairwise adjacency matrix for an unsigned network.

### gRNA design

Guide RNAs (gRNAs) were generated using a commercially available two-piece system termed ALT-R™ CRISPR/Cas9 Genome Editing System (IDT DNA, Coralville, IA). This system combines a custom CRISPR RNA (crRNA) for genomic specificity with an invariant trans-activating RNA (tracrRNA) to produce gRNAs (86). crRNAs were designed using the computational program CCTop/CRISPRator (87, 88), which gauges candidate gRNAs for efficiency and specificity. Each crRNA was annealed separately with tracrRNA in a 1:2 M ratio then combined into a single solution for each gene.

Four gRNAs were used to target each of the ethanol-responsive lncRNA genes Pitt1, Pitt3, and Pitt4 and six gRNAs for Pitt2 (see Supplementary Table S1 for gRNA target sequences). These specifically designed gRNAs bind within a 598, 796, 341, or 372 base pairs (bp) target region that includes the putative promoter and first exon of Pitt1-Pitt4, respectively. We followed the annotations available at the time on the Ensembl Genome Browser (GRCm38/mm10).

### CRISPR/Cas9 mutagenesis

Female C57BL/6J mice were superovulated with 0.1 ml of CARD HyperOva (CosmoBio, #KYD-010) between 10 and 11 AM, followed by 100 IU of human chorionic gonadotropin (Sigma, #CG10) 46–48 h later. Donor females were caged overnight with C57BL/6J males starting 4–6 h post-gonadotropin injection and allowed to mate. Embryos were harvested from oviducts between 9 and 10 AM the following morning, cumulus cells were removed using hyaluronidase, and embryos were cultured under 5% CO_2_ in KSOM medium (Cytospring, #K0101) for 1–2 h. Embryos were electroporated in 5 µL total volume of Opti-MEM medium (ThermoFisher, #31985088) containing 100 ng/μL of each gRNA cocktail and 200 ng/μL Alt-R^®^ S.p. HiFi Cas9 Nuclease V3 protein (IDT, #1081060) with a Bio-Rad Gene-Pulser Xcell in a 1 mm-gap slide electrode (Protech International, #501P1-10) using square-wave pulses (five repeats of 3 msec 25V pulses with 100 msec interpulse intervals). Electroporated embryos were placed back into culture under 5% CO_2_ in KSOM. For *in vitro* validation of Pitt1-Pitt4 gRNAs, embryos were cultured for 3 days until the morulea/blastocyst stage and subsequently analyzed for mutations. For *in vivo* cohort generation, one- or two-cell embryos were surgically implanted into the oviducts of plug-positive CD-1 recipients (20–40 embryos per recipient) that had been mated to vasectomized males the previous night.

### Genotyping

DNA was amplified from individual Pitt1-Pitt4 gRNA-electroporated embryos using a Qiagen Repli-G kit (Qiagen, #150025). DNA was isolated from ear snips of Pitt1-Pitt4 TAKO offspring using Quick Extract (Lucigen, #QE09050). DNAs were genotyped by PCR under the following settings: 95°C for 5 min (1x); 95°C for 30 s, 60°C for 30 s, 72°C for 1 min (40x); 72°C for 10 min (1x). Primers for PCR amplification of Pitt1-Pitt4 are listed in Supplementary Table S1. PCR amplicons of Pitt1-Pitt4 [Wild-type (WT): 929, 963, 581 and 583 bp, respectively] were analyzed by agarose gel electrophoresis.

### RNA preparation

Hippocampal brain tissue from Pitt1-Pitt4 mice was used for RT-PCR analysis. All mice were 16–20 weeks of age at time of euthanasia. Total RNA was isolated using TRIzol (Invitrogen, #15596018) according to the manufacturer’s protocol, and DNA contamination was removed with a TURBO DNA-*free*™ Kit (Invitrogen, #AM1907). Total RNA was analyzed for purity and concentration using a Nanodrop Spectrophotometer (Thermo Scientific, Waltham, MA). One microgram of purified RNA was converted into cDNA using Superscript™ III First-Strand Synthesis System (Invitrogen, #18080051) with random hexamer primers. RT-PCR primers were used that span both the mutation site as well as the downstream probe-binding exonic region for Pitt1-Pitt4 (Supplementary Table S1). A reaction that lacked reverse transcriptase was used as a negative control for each sample tested.

### Behavioral testing

All mice were moved into a reverse light-cycle housing/testing room (lights off at 10 AM) at 5 weeks of age and allowed to acclimate for 2–3 weeks before the start of experiments. Mice were weighed weekly during behavioral experimentation. Ethanol-drinking experiments were performed in the housing room. Mice were singly-housed for all behavioral studies. Mice were sequentially tested on DID and EOD-2BC, with a minimum of 7 days between assays.

Pitt1 and Pitt2 were studied together with a purchased control group (controlled for age, sex, and strain) previously shown to be comparable to mock-treatment controls (83). Similarly, Pitt3 and Pitt4 were studied together with a separate purchased control group.

### One-bottle drinking in the dark

Mice were given access to ethanol (20% v/v) in 15 ml drinking bottles with 3.5-inch sipper tubes (Amuza, San Diego) 2 h into the dark-cycle for 2 consecutive days. Fresh ethanol solution was prepared daily. The first day’s training session lasted for 2 h. The second day’s experimental session lasted 4 h. The amount of ethanol consumed by each mouse was recorded. Empty cages with sipper bottles only were used to control for sipper tube leakage, and leakage amount was subtracted from amount of ethanol consumed by the mice. Immediately following the experimental session, blood samples were collected from tail nicks and the plasma isolated. An Analox analyzer was used to measure the blood ethanol concentrations (BECs) of each mouse (mg/dL; 5 μL).

The Pitt1/Pitt2/control cohorts were assayed based on genotype and not sex (i.e., the Pitt1 TAKOs were assayed separately from the Pitt2 TAKOs). The Pitt3/Pitt4/control cohorts were assayed based on sex and not genotype (i.e., the male Pitt3 and Pitt4 TAKOs were assayed separately from the female Pitt3 and Pitt4 TAKOs).

### Every-other-day two-bottle choice drinking

Mice were given access to ethanol (v/v; ramping every-other-day from 3%, 6%, 9%, 12% until 15% was reached then maintained for a total of 12 days at 15%) and water for 24-h sessions every other day. If a 20% difference from controls in ethanol consumption was not observed at 15% ethanol, then the concentration was increased to 20% v/v and the experiment extended an additional 12 days. Water alone was offered on off days. The side placement of the ethanol bottles was switched with each drinking session to avoid side preference. Bottles were weighed before placement and after removal from the experimental cages. Empty cages with sipper bottles only were used to control for fluid leakage, and leakage amount was subtracted from the amount consumed by the mice. The quantity of ethanol consumed, and total fluid intake was calculated as g/kg body weight per 24 h. Preference was calculated as amount ethanol consumed divided by total fluid consumed per 24 h. Ethanol drinking results were transformed to reflect the percent change in ethanol consumption compared to control. Ethanol solutions were prepared fresh daily.

### Preference for non-ethanol tastants

When a significant difference in ethanol consumption was observed between genotypes, mice were subsequently tested for saccharin (sweet tastant; Sigma-Aldrich, 240931) and quinine (bitter tastant; Sigma-Aldrich, 145912) preference using a 24-h Two-Bottle Choice (2BC) paradigm. One sipper bottle contained the tastant solution and the other contained water. Mice were offered two concentrations of saccharin (0.03% and 0.06%) and quinine (0.03 and 0.06 mM). For each tastant, the lower concentration was presented first followed by the higher concentration. Each concentration was presented for 2 days (4 days total) with at least 7 days of water-only between tastants. Empty cages with sipper bottles only were used to control for leakage, and leakage amount was subtracted from the amount consumed by the mice. Fresh tastant solution was prepared daily.

### Statistical analysis

Statistical analysis was performed using GraphPad Prism (GraphPad Software, Inc., La Jolla, CA). Two-way ANOVA with multiple comparisons was used for Pitt1, Pitt2, and control DID and BEC data, and one-way ANOVA with multiple comparisons was used for Pitt3, Pitt4, and control DID and BEC data. Two-way mixed-effects ANOVA with multiple comparisons and repeated measures was used for Pitt1, Pitt2, and control weight over time, and two-way ANOVA with multiple comparisons and repeated measures was used for EOD-2BC data and Pitt3, Pitt4, and control weight over time. Significant main effects were subsequently analyzed with Benjamini, Krieger, and Yekutieli two-stage linear step up procedure post-hoc analysis (89). Technical failures were appropriately removed from analysis.

Because of well-known sex differences of C57BL/6J on ethanol consumption in the DID and EOD-2BC assays (90–93), male and female mice were tested on separate days (except for Pitt1/Pitt2/control DID and BEC), and each sex was analyzed separately. Statistical significance was defined as *p* ≤ 0.05 and *q* ≤ 0.05. All data are presented as mean ± S.E.M.

## Results

### Perturbation of the transcriptome following CIEV exposure

**FIGURE 1:**
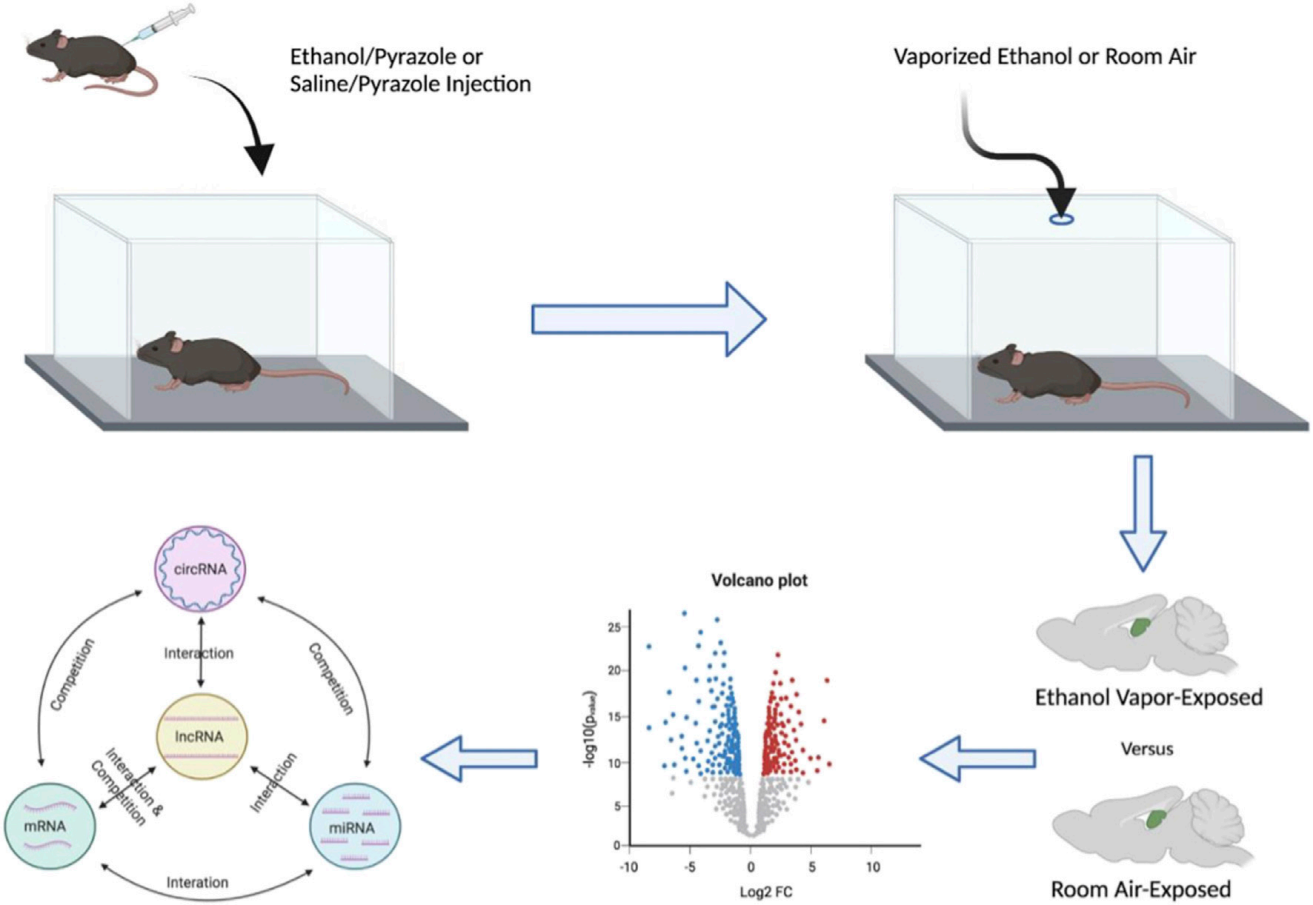
Schematic diagram detailing the experimental pipeline utilized to generate the list of top novel ethanol-responsive hub lncRNA candidates to target for ethanol-related functional interrogation. Male mice were given a priming injection of either ethanol and pyrazole or saline and pyrazole and placed in either an ethanol- or room-air vapor champers for 16 h/day, 4 days/week, for 7 weeks, respectively. Hippocampi were dissected 24 h after the final vapor exposure and then subject to mRNA, lncRNA, circRNA, and miRNA microarray analysis. These data sets were then used to generate ceRNA networks of ethanol-responsive RNA genes.

**FIGURE 2:**
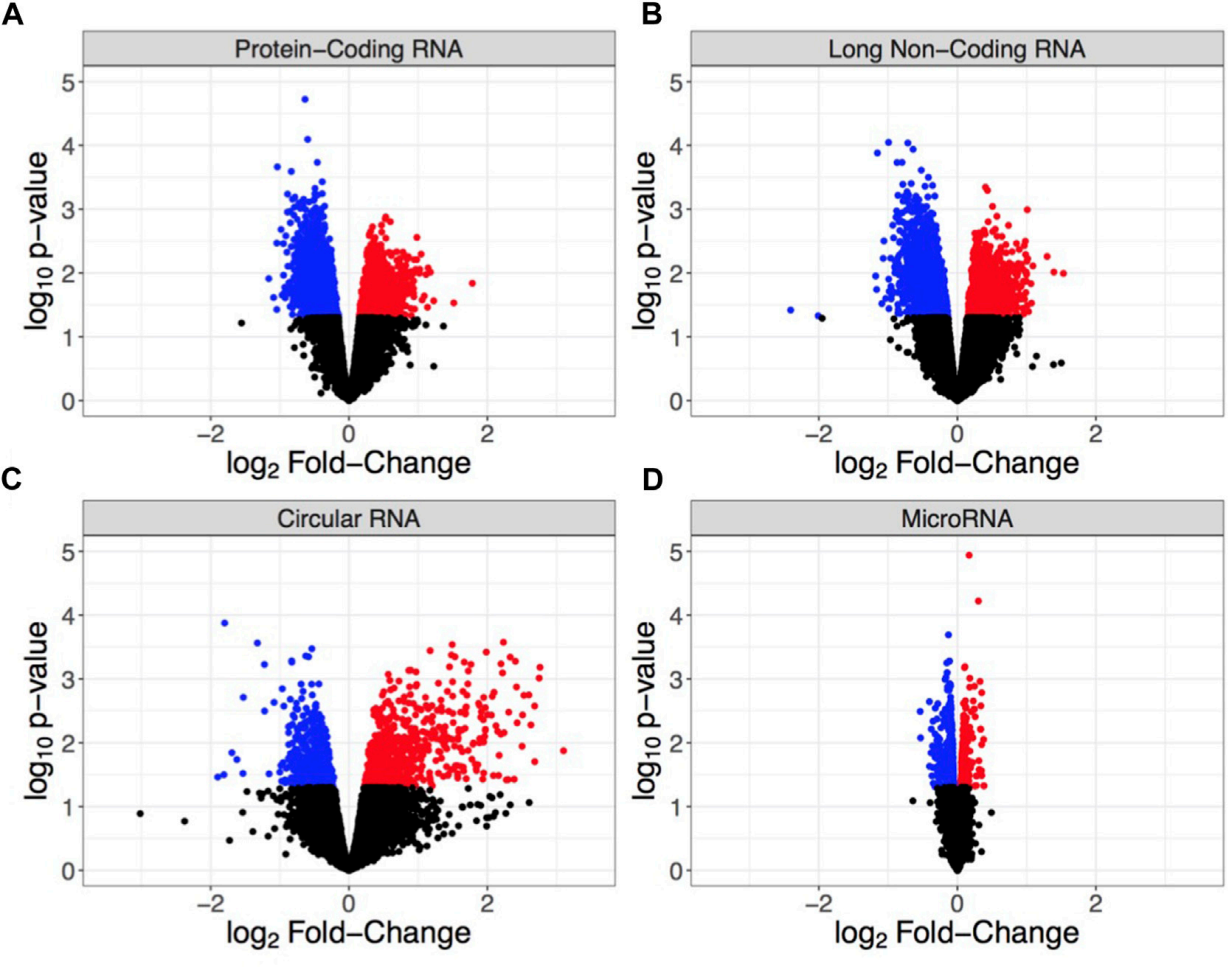
Volcano plots showing differential RNA expression based on log_2_ fold-change in expression (x-axis) and log_10_ *p*-value (y-axis) for **(A)** protein-coding RNA (mRNA), **(B)** long non-coding RNA (lncRNA), **(C)** circular RNA (circRNA), and **(D)** microRNA (miRNA). Each point indicates an individual non-duplicated probe on the microarray with blue = significantly down-regulated, red = significantly up-regulated, and black = non-significant. Significance is defined by *p* < 0.05.

Hippocampi were dissected from male mice chronically exposed to ethanol vapor (CIEV) or room air control for 16 h/day, 4 days/week, for 7 weeks, 24 h after the final vapor exposure. The first 24 h of withdrawal from alcohol is a critical window of time associated with relapse, which can be highly detrimental to the long-term goal of reduced drinking (16). This hippocampal tissue originated from the sires previously described in (84) wherein males maintained BECs ranging from 100 to 250 mg/dl throughout the experiment. Total RNA was isolated from hippocampi for transcriptome analysis to identify biological systems affected by chronic ethanol exposure (Figure 1). We detected a total of 18,283 mRNA probes, 27,177 lncRNA probes, 14,182 circRNA probes, and 23,386 miRNA probes on the microarray. To identify RNAs differentially expressed due to CIEV, our analysis separately examined statistically significant changes (*p* < 0.05) in expression for mRNA, lncRNA, circRNA, and miRNA. Among these four classes of RNAs we found that lncRNAs showed the largest number of changes in expression due to chronic ethanol exposure (*n* = 1,923 up-regulated, *n* = 2,694 down-regulated). This was followed by mRNA (*n* = 1,948 up-regulated, *n* = 2,121 down-regulated), circRNA (*n* = 750 up-regulated, *n* = 729 down-regulated), and miRNA (*n* = 481 up-regulated, *n* = 723 down-regulated) (Figure 2). This data may suggest that a large number of different RNA within the hippocampus are susceptible to chronic ethanol exposure; however, each of these RNA biotypes do not exist in isolation and must work in concert for homeostatic function of cellular systems.

The expression of different RNA subtypes creates tightly coordinated ceRNA networks to mediate the biological function of molecular circuits (76–81) (Figure 1). We used WGCNA to determine the pairwise correlation of RNA expression across samples and assess the total connectivity of lncRNA, mRNA, circRNA, and miRNA. Due to the known biological roles in the regulation of gene expression and their perturbation by chronic ethanol exposure, our analysis focused on identifying ethanol-responsive lncRNAs for *in vivo* characterization. Our unbiased transcriptome analysis determined that there were multiple ethanol-responsive lncRNAs that are present in the GRCm38/mm10 mouse genome assembly but have yet to be characterized for molecular or behavioral function. To determine suitable lncRNAs for follow-up *in vivo* studies, we used a summed rank of lncRNAs based on their statistical significance (*p* < 0.05), fold-change in up-regulation of expression, overall level of expression to focus on the most abundant lncRNAs, and lncRNAs with the highest total connectivity within the correlation networks to concentrate on hubs of coordinatedly regulated RNA expression. Additionally, lncRNAs were screened for the capacity to easily create CRISPy TAKO mice by identifying candidates within intergenic regions that did not overlap any other known genes or regulatory regions in the GRCm38/mm10 mouse genome. Based on this selection criteria the top 4 candidate lncRNA selected for testing were *Gm42575*, *4930413E15Rik*, *Gm15767*, and *Gm33447* (Table 1).

**TABLE 1:** Bioinformatic data of the top-ranked lncRNA genes identified from the ceRNA networks in order.

Name	Probe	Gene symbol	Chromosome	Strand	Start	End	log fold-change	Mean expression	*p*-value
Pitt1	ASMM10P031898	*Gm42575*	chr5	+	74754373	74754432	0.35	9.71	0.03
Pitt2	ASMM10P032341	*4930413E15Rik*	chr5	+	118961191	118961250	0.28	8.82	0.02
Pitt3	ASMM10P034032	*Gm15767*	chr6	−	147242527	147242586	0.27	9.27	0.03
Pitt4	ASMM10P010493	*Gm33447*	chr13	+	97380367	97380426	0.35	8.25	0.02

Given name, probe, gene symbol, chromosome, strand, gene start, gene end, log fold-change, mean expression, and *p*-value are presented.

### CRISPy TAKOs–Pitt1 and Pitt2

#### CRISPR/Cas9-mediated mutagenesis

**FIGURE 3:**
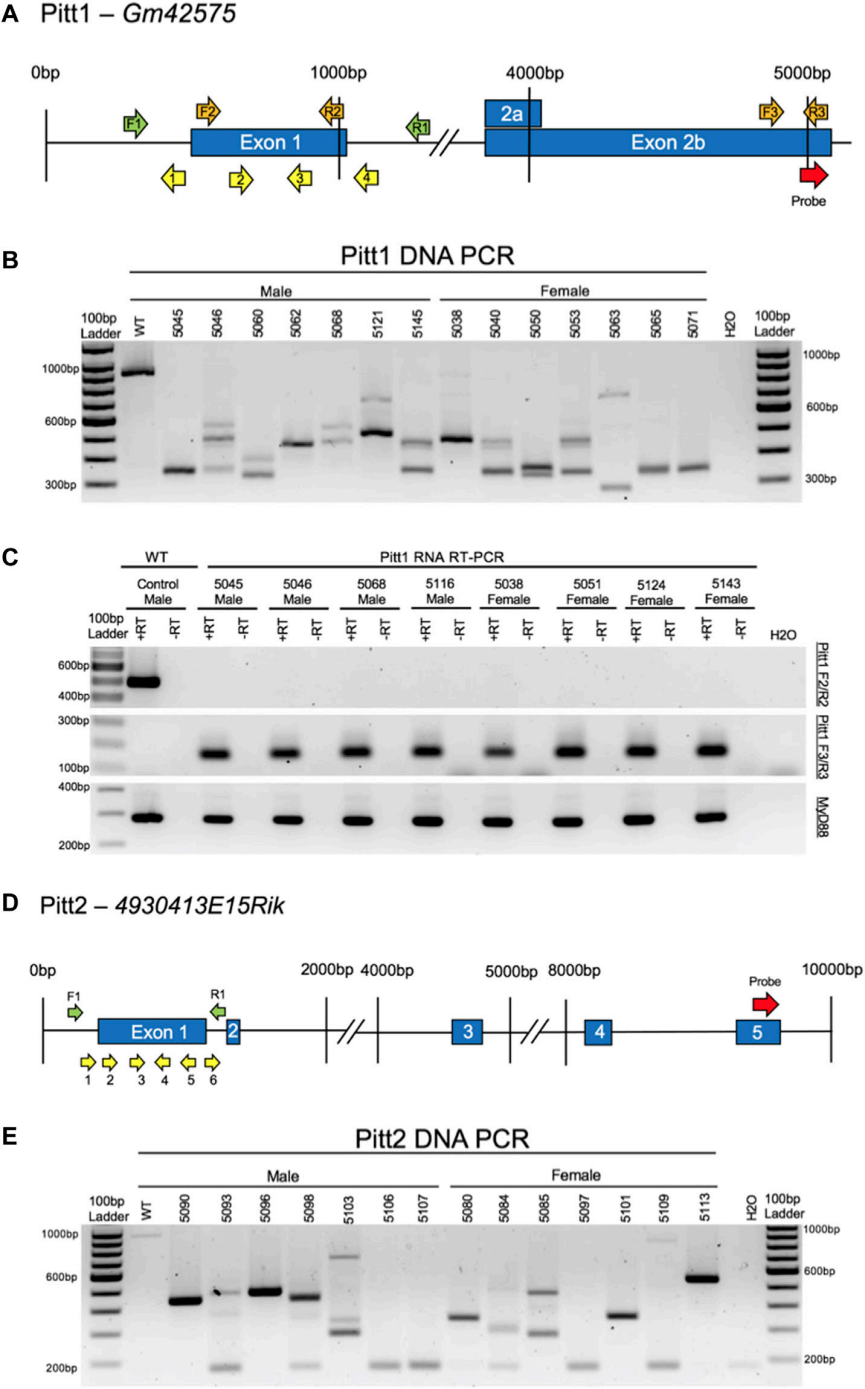
CRISPy TAKO schematics and genotypes for Pitt1 and Pitt2. **(A)** Pitt1 gene symbol and structure. The gRNAs, PCR primers, RT-PCR primers, and probe binding site are shown as yellow, green, orange, and red arrows, respectively. **(B)** Agarose gel electrophoresis of PCR amplicons of Pitt1 DNA in a random representative subset of Pitt1 TAKOs demonstrating abnormal amplicons in TAKO mice compared to WT control. Individual mouse numbers are presented above the gel. **(C)** Random representative subset RT-PCR results from Pitt1 hippocampal brain tissue showing abnormal RNA transcripts. (Top) RT-PCR of Pitt1 exon 1 amplicons using the F2/R2 primers demonstrating abnormal RNA transcripts in TAKO mice compared to WT control. (Middle) RT-PCR amplicons using the F3/R3 primers spanning downstream Pitt1 exons, demonstrating abnormal RNA products in Pitt1 mutant TAKOs that are not present in WT. (Bottom) RT-PCR of *MyD88* amplicons used as an internal control. **(D)** Pitt2 gene symbol and structure. The gRNAs, PCR primers, and probe binding site are shown as yellow, green, and red arrows, respectively. **(E)** Agarose gel electrophoresis of PCR amplicons of Pitt2 DNA in a random representative subset of Pitt2 TAKOs demonstrating abnormal amplicons in TAKO mice compared to WT control. Individual mouse numbers are presented above the gel.

To enhance CRISPR mutagenesis frequency as previously described (83), all lncRNA genes were targeted simultaneously with 4–6 gRNAs tiled 50–200 bp apart from each other, spanning the putative promoter and first exon of each gene. Four gRNAs were designed to span a 598 bp range within the Pitt1 gene (Figure 3A). Six gRNAs were designed to span a 796 bp range within the Pitt2 gene (Figure 3D).

Pitt1 and Pitt2 gRNAs were validated for efficient mutagenesis by analyzing *in vitro* cultured embryos following electroporation. Agarose gel electrophoresis of PCR amplicons that span the targeted locus of Pitt1 and Pitt2 indicated that 100% of embryos harbored indels of various sizes (Supplementary Figures S1A,B, respectively).

A cohort of 35 Pitt1 offspring and 42 Pitt2 offspring, all on the C57BL/6J genetic background, were generated using the CRISPy TAKO approach. All mice born from electroporated embryos were genotyped for gross indels using PCR. The Pitt1 929 bp WT PCR amplicon was readily apparent in control WT DNA but only 2 out of 35 Pitt1 animals (data not shown). The remaining 33 displayed gross indels encompassing the targeted region of interest. PCR bands from a random representative subset of Pitt1 mice selected for behavioral experimentation is shown in Figure 3B. The Pitt2 963 bp WT PCR amplicon was readily apparent in the WT control and 2 out of 42 Pitt2 animals (data not shown). The remaining 40 displayed gross indels encompassing the targeted region of interest. PCR bands from a random representative subset of Pitt2 mice selected for behavioral experimentation is shown in Figure 3E.

The indels varied from animal to animal and most appeared to be deletions, as evidenced by the PCR products being ∼50–400 bp smaller than the 929 bp WT amplicons for Pitt1, and ∼50–600 bp smaller than the 963 bp WT amplicons for Pitt2 (Figures 3B,E, respectively). Out of the 35 Pitt1 mice and 42 Pitt2 mice, only a subset (*n* = 11M/14F Pitt1; 16M/12F Pitt2) harboring a large mutation(s) spanning the putative promoter and exon 1 of Pitt1 or Pitt2 were selected for behavioral phenotyping. It should be noted that the mice used for phenotyping presented variable deletions mainly ranging in 230–730 bp (Figures 3B,E, respectively). Despite all Pitt1 and Pitt2 mice showing variability in mutation site and size, all mice within a genotype were expected to manifest the same effect on gene expression and behavioral phenotypes [as previously shown (83)].

We have previously demonstrated that control C57BL/6J mice purchased from Jackson Laboratories are not significantly different from in-house generated Mock-treatment control mice (83). Therefore, Pitt1 and Pitt2 TAKO mice were compared to age and sex-matched C57BL/6J controls. Mice were weighed once per week during behavioral experimentation. Both TAKO cohorts for both sexes had significantly increased weight compared to controls. Males and females had an effect of genotype [F (1.715, 7.717) = 87.22; *p* < 0.0001] and [F (1.626, 9.758) = 89.44; *p* < 0.0001], respectively (Supplementary Figure S2). Post-hoc analysis revealed an effect of genotype for both Pitt1 and Pitt2 males (*q* < 0.001), and Pitt1 and Pitt2 females (*q* < 0.0001). These results are consistent with previously observed differences in our laboratory in purchased versus in-house produced offspring (data not shown).

#### RNA analysis

Hippocampal RNA from a subset of mutant mice used for phenotyping was analyzed by RT-PCR to validate that the DNA mutations surrounding the putative promoter and first exon of Pitt1 and Pitt2 disrupted expression of the targeted genes. Two RT-PCR primer sets were used for each genotype to characterize the RNA transcript in TAKO versus WT hippocampal RNA. F2/R2 RT-PCR primers were used to validate KO of RNA at the mutation site. F3/R3 RT-PCR primers were used to characterize the downstream exon containing the microarray probe-binding site to investigate expression of downstream lncRNA sequences (Figures 3A,D, respectively).

Pitt1—The top panel of Figure 3C demonstrates that the targeted exon 1 region is not transcribed in Pitt1 TAKOs. The middle panel highlights that the mutation(s) modulate the downstream lncRNA transcript, resulting in expression of a novel transcript that is not observed in the WT control. The bottom panel targeting *MyD88* was used as an internal control.

Pitt2—Despite extensive efforts to produce reliable RT-PCR amplicons for the Pitt2 RNA transcript(s), it was not achievable. RT-PCR amplicons for both the mutation site and probe-binding site of the Pitt2 transcript were inconsistent and variable even in WT control samples (data not shown).

#### Drinking in the dark

**FIGURE 4:**
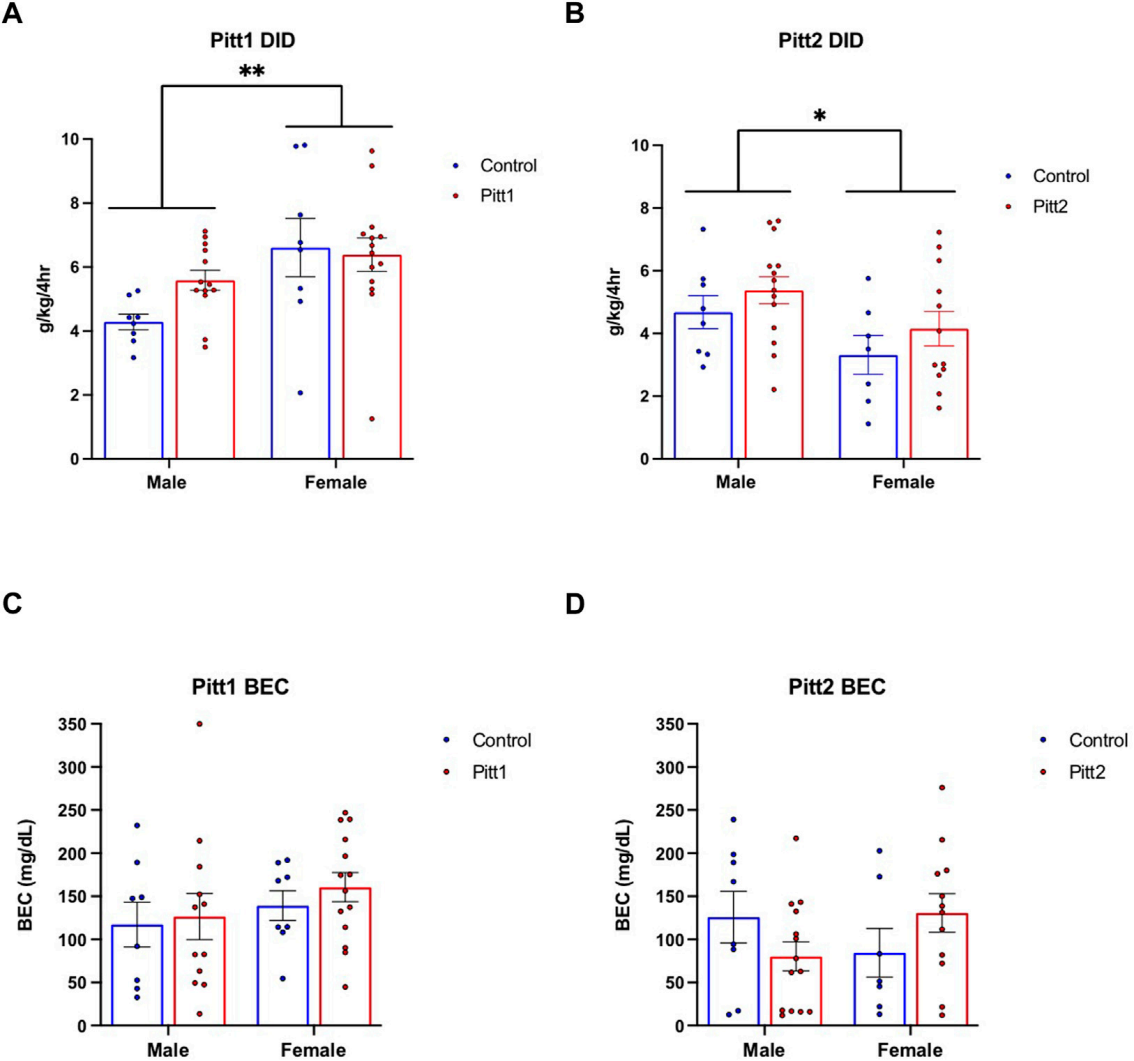
Effect of Pitt1 and Pitt2 mutation on ethanol consumption in the Drinking in the Dark assay. **(A)** Total ethanol consumption of Pitt1 and control mice over a 4-h experimental period (g/kg/4h). N = 13–14 Pitt1 TAKOs; *n* = 8 controls. **(B)** Total ethanol consumption of Pitt2 and control mice over a 4-h experimental period (g/kg/4h). N = 12–14 Pitt2 TAKOs; *n* = 7–8 controls. **(C)** Blood ethanol concentrations (mg/dL; 5 μL) from plasma collected from all Pitt1 mice immediately following removal of ethanol-filled bottles. N = 12–14 Pitt1 TAKOs; *n* = 8 controls. **(D)** Blood ethanol concentrations (mg/dL; 5 μL) from plasma collected from all Pitt2 mice immediately following removal of ethanol-filled bottles. N = 12–14 Pitt2 TAKOs; *n* = 7–8 controls.

Pitt1 and Pitt2 DID data were analyzed separately based on genotype (i.e., Pitt1 males and females were analyzed together with half of the controls, and Pitt2 males and females were analyzed together with the other half of the controls). No statistically significant difference was observed between Pitt1 versus control or Pitt2 versus control for either the 2-h training day (data not shown) or the 4-h experimental day (Figures 4A,B, respectively). Consistently, there was no significant difference between the BECs of Pitt1 and control or Pitt2 and control following the 4-h experimental day for both males and females (Figures 4C,D, respectively). We observed a significant main effect of sex for Pitt1 DID [F (1, 39) = 8.300; *p* < 0.01] where females consumed more ethanol than males. Interestingly, a significant main effect of sex was also observed in Pitt2 DID [F (1, 37) = 5.545; *p* < 0.05], however females unexpectedly consumed less ethanol than the males.

#### Every-other-day two-bottle choice drinking

**FIGURE 5:**
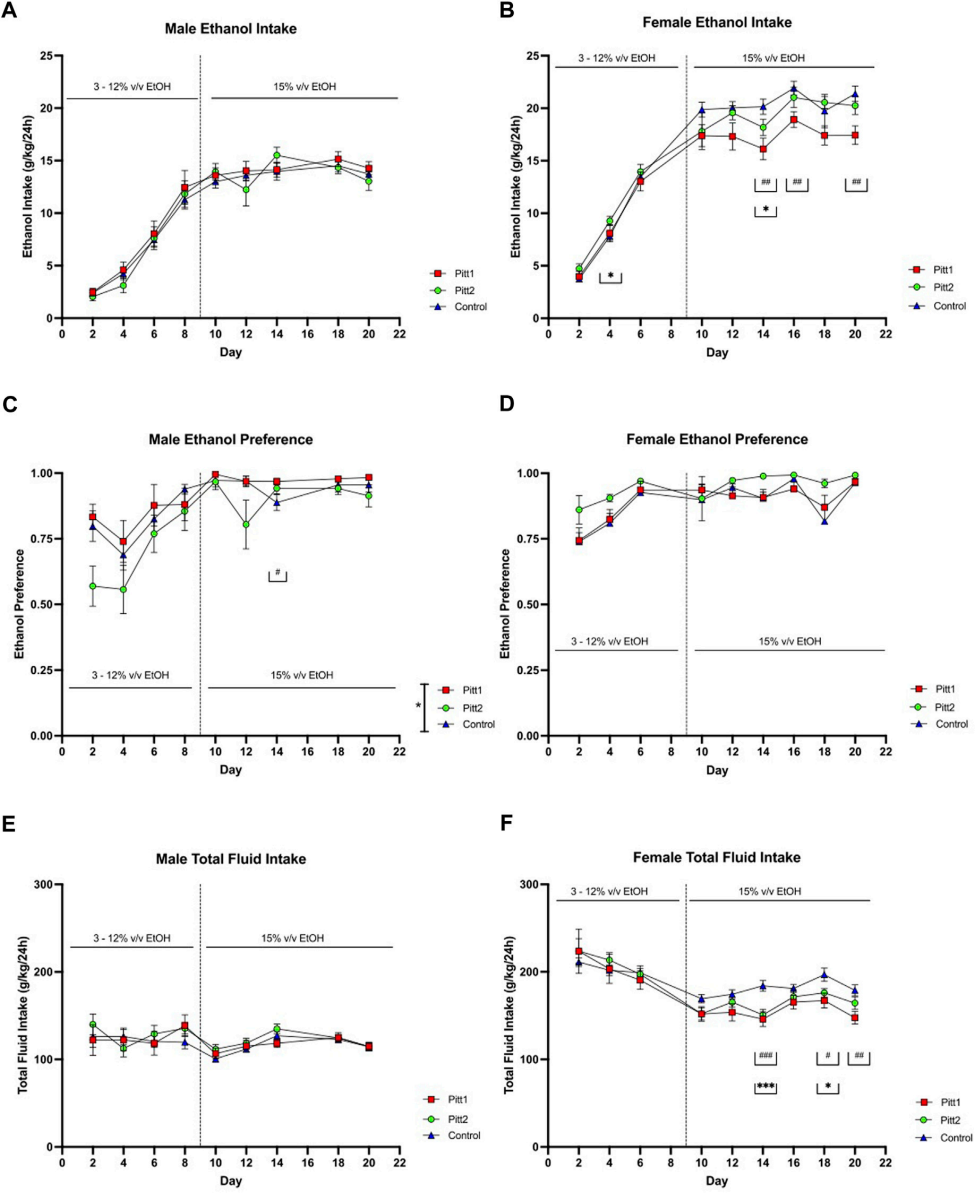
EOD-2BC drinking in Pitt1, Pitt2, and control mice. Left, males; right, females. **(A,D)** ethanol intake (g/kg/24 h), **(B,E)** ethanol preference, and **(C,F)** total fluid intake (g/kg/24 h) in Pitt1 mutant, Pitt2 mutant, and control mice across time and concentration. ^#^ or *q < 0.05, ^##^ or **q < 0.01, and ^###^ or ***q < 0.001 between Pitt1 and control, and Pitt2 and control, respectively. N = 11–16/sex/genotype.

Pitt1, Pitt2, and control mice were tested for ethanol drinking using an EOD-2BC ethanol consumption assay over a period of 20 days. Pitt1, Pitt2 and control male analysis of ethanol intake revealed a main effect of day [F (5.103, 199.0) = 159.5; *p* < 0.0001], but no effect of genotype or day x genotype (Figure 5A). Analysis of ethanol preference in males revealed a main effect of day [F (4.715, 183.9) = 15.83; *p* < 0.0001] and genotype [F (2, 39) = 3.755; *p* < 0.05], but no day x genotype significant differences (Figure 5C). Post-hoc analysis revealed that on day 14 Pitt1 males had significantly higher ethanol preference than control males (*q* < 0.05). Pitt1 male ethanol preference at 15% v/v ranged from 0% to 9% increase, while Pitt2 male ethanol preference ranged from an increase of 6% to a decrease of 17% (Supplementary Figure S3C). For total fluid intake, there was a main effect of day [F (3.508, 136.8) = 4.612; *p* < 0.01] but no effect of genotype or day × genotype interaction for the males (Figure 5E). Due to a record-keeping error, data from day 16, at 15% v/v ethanol, was lost.

Analysis of Pitt1, Pitt2, and control female cohorts on total ethanol intake revealed a day × genotype interaction [F (16, 304) = 2.679; *p* < 0.001] and main effect of day [F (4.409, 167.5) = 286.3; *p* < 0.0001], but no effect of genotype (Figure 5B). Post-hoc analysis revealed that on days 14, 16, and 20 Pitt1 females consumed significantly less ethanol than control (*q* < 0.01), and Pitt2 females consumed significantly more ethanol than control on day 4 (q < 0.05), and significantly less on day 14 (*q* < 0.05). Pitt1 females consistently consumed 10%–20% less ethanol at 15% v/v. Pitt2 females only consumed up to 10% less ethanol at 15% v/v (Supplementary Figure S3B). Analysis of ethanol preference in females revealed a main effect of day [F (3.743, 142.2) = 13.60; *p* < 0.0001], but no effect of genotype or day x genotype (Figure 5D). For total fluid intake, there was a day x genotype [F (16, 304) = 1.938; *p* < 0.01] and main effect of day [F (2.272, 86.32) = 31.91; *p* < 0.0001], but no effect of genotype (Figure 5F). Post-hoc analysis revealed that on days 14, 18, and 20 Pitt1 females consumed significantly less total fluid than control females (*q* < 0.0001, *q* < 0.05, and *q* < 0.01, respectively) and that on days 14 and 18 Pitt2 females consumed less total fluid than control females (*q* < 0.0001 and *q* < 0.05, respectively). The change in ethanol intake coincided with a reduction in total fluid for Pitt1 females at 15% v/v ethanol ranging from a reduction of 8.5%–20.5%, and Pitt2 females ranging from a reduction of 5%–18% (Supplementary Figure S3F). Due to a record-keeping error, data from day 8, at 12% v/v ethanol, was lost. Since the decrease in female ethanol intake could be linked to a reduction in overall fluid intake, and the male data was not highly compelling, the experiment was terminated following the completion of 15% v/v EOD-2BC.

#### Preference for non-ethanol tastants

Changes in taste perception can alter ethanol consumption in mice (94–96). Because female Pitt1 and Pitt2 displayed altered EOD-2BC ethanol consumption compared to controls, females were subjected to both sweet (i.e.*,* saccharin) and bitter (i.e.*,* quinine) tastants. A 24-h 2BC assay was used to determine whether an alteration in taste perception could account for the observed changes in ethanol consumption in the mutant lines tested. No significant difference was observed between genotypes for either saccharin (Supplementary Figure S4A) or quinine preference (Supplementary Figure S4B).

### CRISPy TAKOs–Pitt3 and Pitt4

#### CRISPR/Cas9-mediated mutagenesis

**FIGURE 6:**
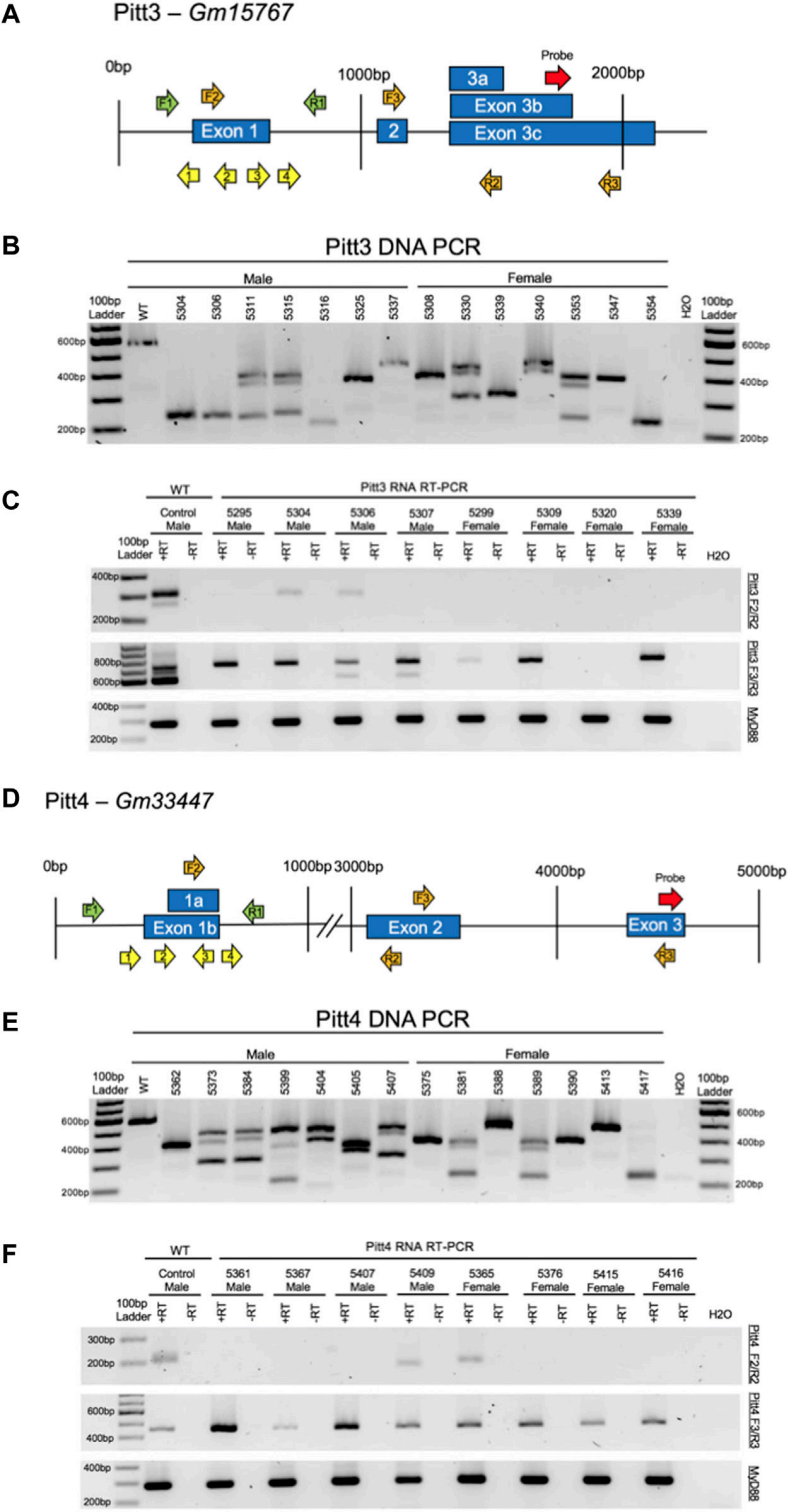
CRISPy TAKO schematics and genotypes for Pitt3 and Pitt4. **(A)** Pitt3 gene symbol and structure. The gRNAs, PCR primers, RT-PCR primers, and probe binding site are shown as yellow, green, orange, and red arrows, respectively. **(B)** Agarose gel electrophoresis of PCR amplicons of DNA from a random representative subset of Pitt3 TAKOs. Individual mouse numbers are presented above the gel. **(C)** Random representative subset of RT-PCR results from Pitt3 hippocampal brain tissue showing abnormal RNA transcripts in TAKO mice compared to WT control. (Top) RT-PCR of Pitt3 exon 1 using the F2/R2 primers demonstrating the absence of the WT amplicon in most mice, although two animals (5304 and 5306) express a WT sized transcript at an apparently reduced level. (Middle) RT-PCR amplicons using F3/R3 primers spanning downstream Pitt3 exons demonstrating abnormal RNA products in Pitt3 mutant TAKOs compared to controls. (Bottom) RT-PCR of *MyD88* used as an internal control. **(D)** Pitt4 gene symbol and structure. The gRNAs, PCR primers, RT-PCR primers, and probe binding site are shown as yellow, green, orange, and red arrows, respectively. **(E)** Agarose gel electrophoresis of PCR amplicons of DNA from a random representative subset of Pitt4 TAKOs. Individual mouse numbers are presented above the gel. **(F)** Random representative subset of RT-PCR results from Pitt4 hippocampal brain tissue showing abnormal RNA transcripts. (Top) RT-PCR of Pitt4 exon 1 amplicons using the F2/R2 primers demonstrating that the mutations eliminate expression of the WT transcript in 7 of 8 Pitt4 TAKOs analyzed. (Middle) RT-PCR amplicons of downstream Pitt4 exons amplified with the F3/R3 primers demonstrating expression of normal sized transcripts in TAKOs compared to WT control. (Bottom) RT-PCR of *MyD88* amplicons used as an internal control.

A second cohort of mice targeting Pitt3 and Pitt4 (Figures 6A,D, respectively) were subsequently characterized and tested for behavior. Initial validation of gRNAs designed to target Pitt3 and Pitt4 occurred *in vitro* using electroporated embryos (Supplementary Figures S1C,D, respectively) and demonstrated that both genes were mutated at a high frequency.

A total of 70 offspring for Pitt3 and 62 offspring for Pitt4 were generated on the C57BL/6J background using the CRISPy TAKO approach. All mice born from electroporated embryos were genotyped for gross indels using PCR and agarose gel electrophoresis. The Pitt3 581 bp WT PCR amplicon was readily apparent in WT control and 9 out of 70 Pitt3 animals (data not shown). The remaining 61 mutants displayed gross indels encompassing the targeted region of interest. The indels from a random representative subset of Pitt3 TAKOs used for behavioral phenotyping varied from animal to animal and most appeared to be deletions, as evidenced by the PCR products being ∼50–350 bp smaller than the 581 bp WT amplicons (Figure 6B). The Pitt4 583 bp WT PCR amplicon was readily apparent in WT control and 4 out of 62 Pitt4 animals (data not shown). The remaining 58 mutants displayed gross indels encompassing the targeted region of interest. The indels from a random representative subset of Pitt4 TAKOs used for behavioral phenotyping demonstrated deletions ranging from ∼50–350 bp smaller than the 583 bp WT amplicon (Figure 6E). Of the Pitt3 and Pitt4 mutant mice produced, a subset (*n* = 15/sex/genotype) harboring large deletions spanning the putative promoter and first exon of Pitt3 or Pitt4 were selected for behavioral phenotyping.

As noted for Pitt1 and Pitt2 cohorts, Pitt3 and Pitt4 males and females consistently weighed significantly more than controls (Supplementary Figure S5). Analysis of male Pitt3, Pitt4, and control weight over time revealed a main effect of day [F (2.477, 104) = 412.1; *p* < 0.0001], a main effect of genotype [F (2, 42) = 19.48; *p* < 0.0001], and day x genotype [F (12, 252) = 3.599; *p* < 0.0001]. Post-hoc analysis for both males and females, for all weeks, had a significant increase in weight compared to control (*q* < 0.0001).

#### RNA analysis

Hippocampal RNA was isolated from a subset of mutant mice used for behavioral phenotyping and analyzed by RT-PCR to validate that the DNA mutations surrounding the putative promoter and first exon of Pitt3 and Pitt4 disrupted expression. Two RT-PCR primer sets were used for each genotype to characterize the RNA transcript in TAKO versus control hippocampal RNA. F2/R2 RT-PCR primers were used to examine RNA at the site of mutation, and F3/R3 RT-PCR primers were used to characterize expression of the downstream exon containing the microarray probe-binding site (Figures 6A,D, respectively).

Pitt3—The top panel of Figure 6C demonstrates that the exon 1 region in the control sample expressed both the expected 303 bp amplicon as well as an unexpected, slightly larger amplicon. These transcripts were not transcribed in 75% of the Pitt3 TAKOs tested. Two of eight mice (25%; 5304 and 5306) still expressed the slightly larger RNA transcript from exon 1, but at an apparently reduced level. The middle panel highlights variability in expression between animals. Some TAKO mice expressed two downstream transcripts (5306 and 5307), some only one transcript (5295, 5304, 5229, 5309, and 5339), and one had no downstream transcripts (5320). This is likely due to variability in deletions of poorly characterized regulatory sequences surrounding the mutation site. The bottom panel targeting *MyD88* was used as an internal control.

Pitt4—The top panel of Figure 6F demonstrates that the targeted exon 1 region was not transcribed in 75% of Pitt4 TAKOs tested. One sample, 5365, still expressed the control-sized transcript, and one sample, 5409, expressed a slightly smaller RNA transcript. This ∼10–20 nt smaller RNA transcript likely reflects an internal mutation that was within the boundaries of the RT-PCR primers. The middle panel revealed that all Pitt4 TAKO mice still produced the downstream Pitt4 transcript, albeit at variable levels of expression. The bottom panel targeting *MyD88* was used as an internal control.

#### Drinking in the dark

**FIGURE 7:**
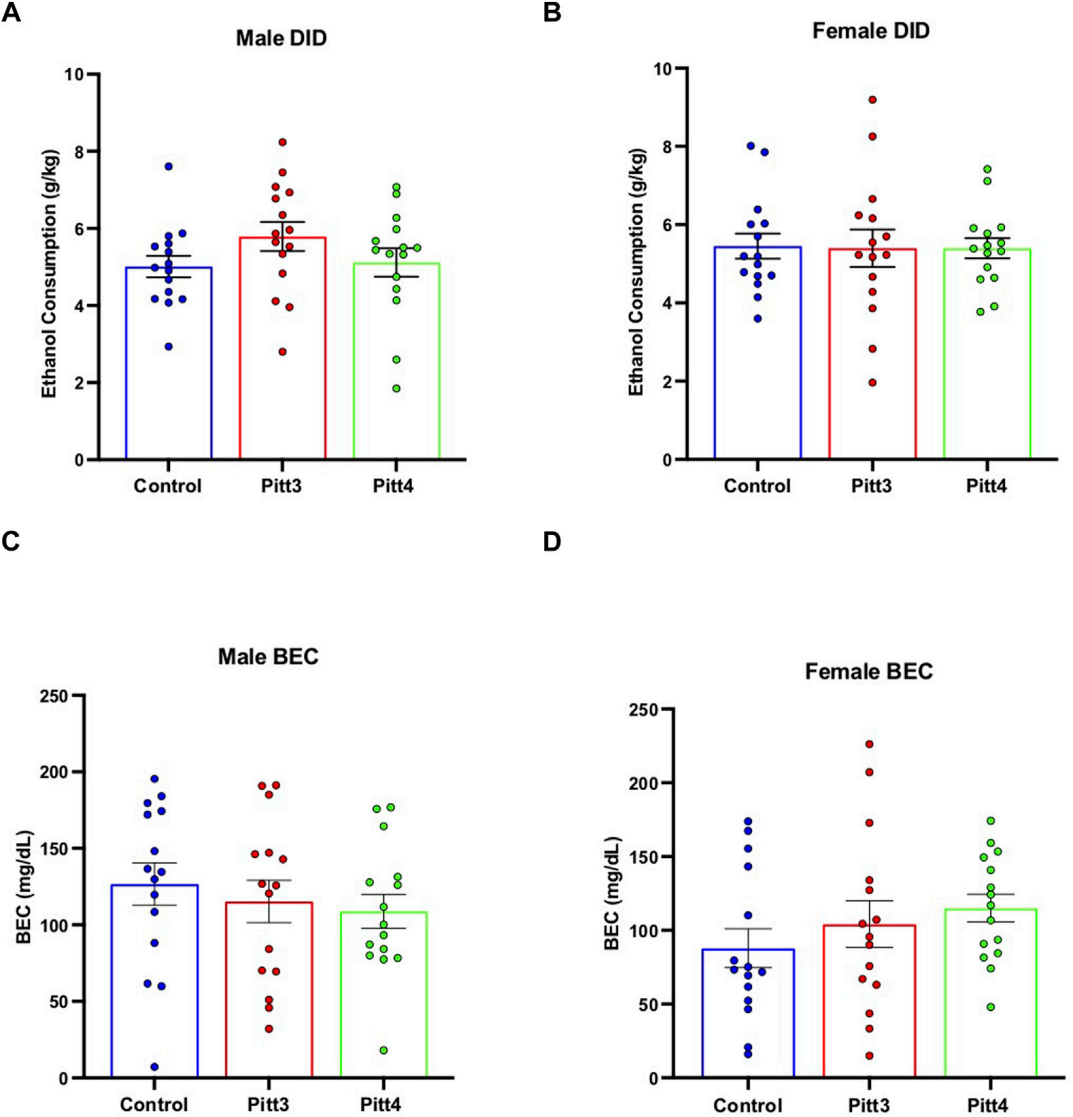
Effect of Pitt3 and Pitt4 mutation on ethanol consumption in the Drinking in the Dark assay. Total ethanol consumption of Pitt3, Pitt4, and control male **(A)** and female **(B)** mice over a 4-h experimental period (g/kg/4h). Blood ethanol concentrations (mg/dL; 5 μL) from plasma collected from all male **(C)** and female **(D)** mice immediately following the removal of ethanol-filled bottles.

Mice were tested for binge-like drinking behavior using the DID ethanol consumption paradigm. Cohorts were separated and analyzed based on sex. No significant difference was observed between Pitt3, Pitt4, and control males (Figure 7A) or females (Figure 7B) for either the 2-h training day (data not shown) or the 4-h experimental day. Consistently, there were also no significant differences between Pitt3, Pitt4, and control male (Figure 7C) or female (Figure 7D) BECs following the 4-h experimental day.

#### Every-other-day two-bottle choice drinking

**FIGURE 8:**
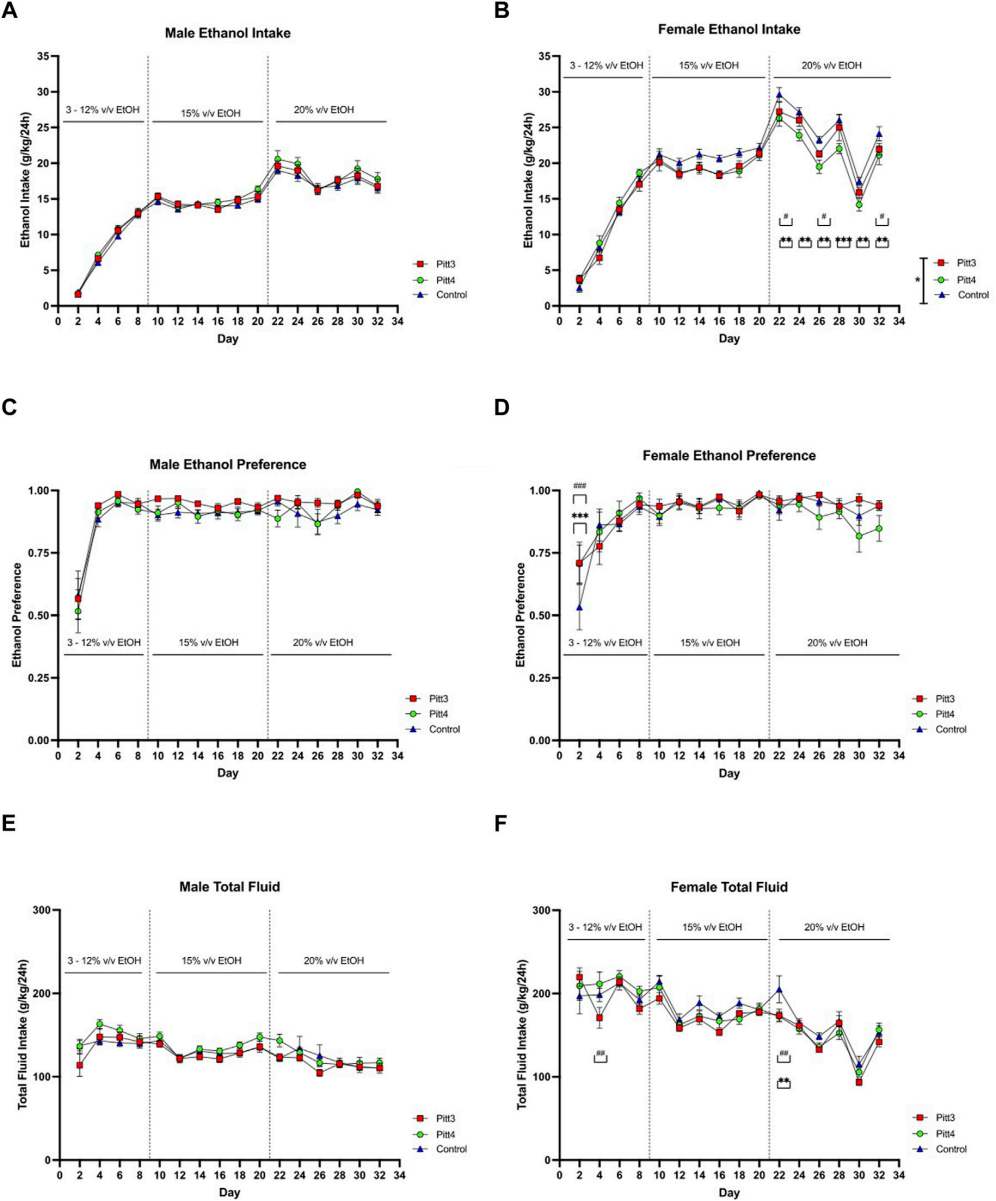
EOD-2BC drinking in Pitt3, Pitt4, and control mice. Left, males; right, females. **(A,D)** ethanol intake (g/kg/24 h), **(B,E)** ethanol preference, and **(C,F)** total fluid intake (g/kg/24 h) in Pitt3 mutant, Pitt4 mutant and control mice across time and concentration. Values represent Mean ± SEM. ^#^ or *q < 0.05, ^##^ or **q < 0.01, and ^###^ or ***q < 0.001 between Pitt3 and control, and Pitt4 and control, respectively).

Pitt3, Pitt4, and control mice were tested for ethanol drinking using an EOD-2BC ethanol consumption assay. Because this set of TAKO animals did not present a significant difference in total fluid intake following 15% v/v ethanol, the experimental paradigm was expanded to include 20% v/v ethanol. Analysis of male Pitt3, Pitt4, and control ethanol intake revealed a main effect of day [F (15, 625) = 335.2; *p* < 0.0001], but no effect of genotype or day x genotype (Figure 8A). Analysis of male ethanol preference revealed a main effect of day [F (15, 624) = 39.54; *p* < 0.0001], but no effect of genotype or day x genotype (Figure 8C). Consistently, analysis of male total fluid revealed a significant main effect of day [F (15, 624) = 19.39; *p* < 0.0001], but no effect of genotype or day x genotype (Figure 8E).

Analysis of ethanol intake in Pitt3, Pitt4, and control females revealed significant main effects of genotype [F (2, 42) = 3.302; *p* < 0.05], day [F (15, 630) = 248.6; *p* < 0.0001], and a day x genotype [F (30, 630) = 2.201; *p* < 0.001] (Figure 8B). Post-hoc analysis revealed that on day 22, 26, and 32 Pitt3 females consumed significantly less ethanol than controls (*q* < 0.05). On days 22–32 Pitt4 females consumed significantly less than control females (*q* < 0.01, *q* < 0.01, *q* < 0.01, *q* < 0.001, *q* < 0.01, and *q* < 0.01, respectively). Pitt3 females at both 15% and 20% v/v ethanol consumed up to 10% less ethanol compared to control. Pitt4 females consumed up to 12% less at 15% v/v and reached a reduction of up to 18.5% at 20% v/v ethanol. Interestingly, both Pitt3 and Pitt4 females consumed ∼50% more ethanol at 3% v/v (Supplementary Figure S6B). Analysis of female ethanol preference revealed a significant main effect of day [F (15, 630) = 19.28; *p* < 0.0001] and day x genotype [F (30, 630) = 1.596; *p* < 0.05], but no effect of genotype (Figure 8D). Post-hoc analysis revealed a significant increase in ethanol preference compared to control on day 2 for both Pitt3 and Pitt4 (*q* < 0.001). Both Pitt3 and Pitt4 females had a preference ranging from 0–10% difference from control at 15% and 20% v/v ethanol, with ∼35% increase at 3% v/v (Supplementary Figure S6D). Considering total fluid intake in females, there was a significant main effect of day [F (15, 630) = 43.97; *p* < 0.0001] and day x genotype [F (30, 630) = 1.542; *p* < 0.05], but no effect of genotype (Figure 8F). Post-hoc analysis revealed that on day 4 Pitt3 females consumed significantly less total fluid than control females (*q* < 0.01) and on day 22 both Pitt3 and Pitt4 females consumed significantly less total fluid than control females (*q* < 0.01). Both Pitt3 and Pitt4 females had reductions in total fluid intake by up to 19% in Pitt3 and 16% in Pitt4 females at 20% v/v ethanol (Supplementary Figure S6F).

#### Preference for non-ethanol tastants

Since Pitt3 and Pitt4 females had altered EOD-2BC ethanol consumption when compared to controls, females were subject to both sweet (i.e., saccharin) and bitter (i.e., quinine) tastant preference analysis. No differences were observed between genotypes for saccharin preference (Supplementary Figure S7A). For quinine preference, there was a significant main effect of day [F (3, 126) = 3.444; *p* < 0.05], but no main effect of genotype or day x genotype (Supplementary Figure S7B).

## Discussion

Identification of phenotypically relevant ethanol-responsive regulatory genes that control brain transcriptional networks offer valuable insight into the chronic effects of ethanol exposure and AUD. Microarray analysis of hippocampal RNA from male mice exposed to CIEV was used to discern ceRNA expression networks that included four prominent RNA subtypes: lncRNA, mRNA, circRNA, and miRNA (Figure 1). The top four ethanol-responsive hub lncRNAs were identified and selected for functional interrogation. These novel lncRNAs, named Pitt1-Pitt4, interact and compete with a myriad of transcripts to modulate specific ceRNA networks. We hypothesized that directly altering the expression of these lncRNAs would change downstream biological processes and change ethanol-related drinking behavior. Cohorts of Pitt1-Pitt4 gene KO mice were created using the CRISPy TAKO method (83) and subsequently screened for changes in ethanol drinking using the DID and EOD-2BC drinking assays. We observed female-specific reductions in ethanol consumption ranging from 10%–20% in the EOD-2BC paradigm compared to control in three of the tested Pitt mutant lines; Pitt1, Pitt3, and Pitt4. Some of the observed changes were associated with reductions in total fluid consumption but they were not influenced by a change in taste perception. No changes in binge-like drinking in the DID paradigm were observed in either the male or female mutants for any Pitt TAKO genotype (Table 2).

**TABLE 2:** Summary table of behavioral results.

Behavior	M	M	M	M	F	F	F	F
	Pitt1	Pitt2	Pitt3	Pitt4	Pitt1	Pitt2	Pitt3	Pitt4
DID and BEC	No	No	No	No	No	No	No	No
Ethanol Intake	No	No	No	No	Yes (−20%–6%)	Yes (−10%–26%)	Yes (−18%–49%)	Yes (−19%–48%)
Ethanol Preference	Yes (−6%–9%)	Yes (−28%–6%)	No	No	No	No	Yes (−10%–33%)	Yes (−10%–33%)
Total Fluid	No	No	No	No	Yes (−21%–6%)	Yes (−18%–6%)	Yes (−19%–11%)	Yes (−16%–6%)

Words in red represent unchanged behaviors, words in green represent changed behaviors.

The CRISPy TAKO approach was utilized to rapidly generate a cohort of mutant animals in a single generation (83). This offers a quick approach to functionally screen novel lncRNAs of interest so the genes can be quickly tested for the ability to alter behavior, saving both time and resources. This is important when screening large numbers of genes with unknown function for ethanol-related behaviors and avoids the bottleneck of standard reverse-genetic approaches. Electroporating embryos with 4–6 gRNAs targeting a >1 kb region led to unique mutations from the various combinations of gRNAs in each animal produced (83). Those harboring desirable large mutations in their DNA were selected for behavioral experimentation, producing a cohort of uniquely mutated mice in one generation, all hypothesized to interfere with gene function (83).

### RNA analysis

Hippocampal RNA was analyzed by RT-PCR to confirm that mutation of the putative promoter and first exon of each lncRNA gene disrupted gene expression from each targeted locus. Using primers that bind to the putative first exon (Pitt1 and Pitt3) or exon 1 and exon 2 (Pitt4) we established that the CRISPy TAKO mutagenesis approach successfully disrupted gene expression of the targeted loci. Nearly all animals failed to amplify with these primer sets. It should be noted that Pitt4 5365 was the only mouse to express transcripts that appeared like WT, but likely at a reduced level of expression (Figure 6F; top panel). The other Pitt4 mouse, 5409, expressed a slightly smaller transcript than WT, suggesting that an internal mutation within the boundaries of the RT-PCR primers may have been retained, or an alternate splice variant was expressed.

Each hippocampal RNA sample was also analyzed with RT-PCR using primers targeting the probe-binding exon used for the initial microarray analyses that identified these lncRNAs, downstream from the mutation site. This was conducted to determine if the full transcript had been knocked out, or if downstream sequences were still transcribed following mutagenesis of the putative promoter and first exonic region. Regions downstream of the Pitt1, Pitt3, and Pitt4 mutations were expressed in the majority of animals. Surprisingly, the Pitt1 downstream amplicon was not detectable in control samples but was consistently expressed in all Pitt1 TAKO mice (Figure 3C; middle panel). These results are likely due to mutation of the putative promoter activating a normally silent promoter, or by altering downstream splicing events. Pitt3 RT-PCR results revealed variable downstream RNA products; of the eight TAKOs used for RT-PCR, two TAKOs express two downstream transcripts (5306 and 5307), five TAKOs express only a single downstream transcript (5295, 5304, 5229, 5309, and 5339), and one TAKO does not express either downstream transcript (5320). Interestingly, none of the Pitt3 TAKOs had similar RT-PCR results compared to WT (Figure 6C; middle panel). As detailed previously, CRISPy TAKO mutants harbor variable mutations (83) and at some loci such as Pitt3, this can lead to expression of novel transcripts from the targeted locus. This could be the result of the mutations impacting the 5’ splice site(s), or mutating splicer enhancer/repressor binding sites and therefore shifting splicing dynamics (97–101). Analysis of downstream sequences in Pitt4 mutants revealed that the downstream cDNA amplicon was readily detected in control and all TAKOs analyzed (Figure 6F; middle panel). The most parsimonious explanation for these results is that an alternate promoter is present that is driving this downstream transcript (102–104).

Unexpectedly, following extensive experimentation, the Pitt2 transcript at the mutation site and probe-binding site were unable to be reliably amplified from either control or Pitt2 TAKO cDNA. This could have occurred due to Pitt2 RNA being expressed at very low levels, or the Pitt2 gene structure could have been inaccurately annotated. These results highlight an important limitation of working with previously unstudied genes including the majority of lncRNAs. Current gene structure annotations may not accurately predict function and unexpected changes in gene expression may be observed when putative regulatory sequences are deleted form the genome.

The RT-PCR data provided a representative look into the potential transcriptome differences between the TAKO mice within a genotype, such as the three different variants of the downstream Pitt3 amplicon(s). Whereas all Pitt1 TAKOs tested produced identical amplicons for both the mutation site and downstream probe-binding region, it is possible that the Pitt3 TAKO mice could be further divided into sub-genotypes based on their retained RNA transcripts and their expression levels. The observed Pitt3 phenotype could be dampened by the variability of transcripts expressed in each TAKO. Variation in behaviors within a mutant line could be the result of small versus large mutations, novel transcripts being produced, altered expression levels of unmutated transcripts, altered or ablated lncRNA functionality, ethanol-responsive versus ethanol-unresponsive variations, or a combination of such molecular events. However, the spread of data points from all genotypes were similar to control and each other; they were well clustered together, suggesting that independent sub-genotypes did not differ in behavior significantly from each other. To discern these intricacies however, Sanger Sequencing, subcloning, and rigorous molecular testing and statistical analysis of the individual animals would be required.

### Behavioral results

Pitt1-Pitt4 female TAKO mice all demonstrated at least a 10% difference from control in ethanol drinking behavior when tested with the EOD-2BC paradigm (Table 2). This includes ∼20% decrease in ethanol consumption in Pitt1 females at 15% v/v ethanol and in Pitt4 females at 20% v/v ethanol. However, the associated reduction in total fluid intake at their respective concentrations could suggest an alternate reason for the ethanol consumption reduction beyond genotype and sex alone. It should be noted, however, that there was no difference found in total fluid intake under the non-ethanol 2BC tastant paradigms for females of all genotypes (data not shown). Large changes in ethanol consumption and/or preference were also observed between mutant lines and controls during the initial ethanol ramping stage (Figures 5, 8). Pitt2, Pitt3, and Pitt4 female mutants all showed increased ethanol consumption ranging from ∼25%–50% on ramping days with 3% and 6% v/v ethanol (Supplementary Figures S3, S6, respectively). While these results at lower ethanol concentrations are intriguing, our primary focus was the impact on the higher-level concentrations of 15% and 20% v/v ethanol. All four of the lncRNAs targeted are capable of modulating ethanol drinking behavior, with Pitt1, Pitt3, and Pitt4 influencing ethanol consumption in a sex-specific manner.

While differences in ethanol intake were readily apparent throughout the EOD-2BC paradigm in all mutant lines, no differences were observed in DID ethanol consumption or the BECs of the animals immediately following DID (Table 2). This could be due to the obvious differences between the short-term binge-like paradigm and the long-term escalation-of-drinking paradigm and suggestive of specific behavioral patterns being altered by mutation of these lncRNAs that only present in one manner of ethanol consumption. The impacted ceRNA networks may function alternatively from control dependent on the paradigm employed, leading to the deviation in drinking behavior over time.

### Sexual dimorphism

Our data supports the identification and partial characterization of four novel ethanol-responsive lncRNAs that can alter ethanol drinking behavior, specifically in females. Sexually dimorphic behavioral responses to ethanol have been previously reported in the literature for alcohol (30, 105–109). LncRNA genes have shown sex-specific expression in reward pathways, cell signaling, structural plasticity, complex decision making, and behaviors (110–112). Sexually dimorphic biology is present in many stages of drug addiction, including acute reinforcement, the transition to compulsive drug use, withdrawal-associated states of negative affect, craving, and relapse (113). Further, there are known differences in neural systems related to addiction and reward behavior such as epigenetic organization, expression, and contingency that are sex-dependent (113). This suggests that lncRNAs may be important in sexually dimorphic biology and behaviors associated with substance misuse.

The female-specific behavioral changes observed in ethanol drinking were somewhat unexpected as the ethanol-regulated lncRNAs studied were identified from microarray data that originated from a male-only cohort. Male samples were used because of tissue availability [hippocampal tissue originated from the sires described in (84)]. The sex differences observed are likely either qualitative and/or based on underlying differences in mechanism(s) of action (113). For example, there may be differences between the sexes in baseline or ethanol-induced expression levels of Pitt1-Pitt4 lncRNAs. To investigate possible expression differences, analogous female tissue would need to be collected, analyzed, and compared to the male microarray data. This would shed light on not only potential differences in Pitt1-Pitt4 expression between sexes and insight into the observed behavior presented, but also would allow for the identification of sex-independent and additional sex-specific genes.

### LncRNAs and conclusion

A handful of studies has already begun to research lncRNAs in relation to the neurobiology of AUD (4, 41, 42, 114–116). The biological functions of these novel ethanol-linked lncRNAs have been associated with altered gene networks and RNA co-expression (114), alternative splicing (4), and neural function (116). The lncRNA *brain-derived neurotrophic factor antisense* has previously been described as a regulator of epigenetic events in the amygdala of humans with AUD (41). Additionally, the lncRNA named *long non-coding RNA for alcohol preference* was identified as a hub gene whose mutation increased alcohol consumption and preference in Wistar rats compared to controls (42). While the field is growing, there are still over 100,000 lncRNA transcripts (45–49) that remain uncharacterized for their relevance to AUD and other human disorders but hold the potential to regulate multiple cellular mechanisms and behaviors.

Mutating these novel uncharacterized Pitt1-Pitt4 lncRNA genes may impact a number of molecular functions, such as subcellular localization, sequestration, scaffolding, and epigenetic regulation of gene expression (44, 50–53). Our study was specifically designed to test genes with no known molecular or behavioral functions related to models for AUD. We conducted these studies with the hypothesis that several, if not all, of the top-ranked genes would have the ability to alter ethanol drinking and provide an ideal candidate gene for more in-depth molecular characterization. By removing a large exonic region of these genes, many different mechanisms of action could have been altered that manifest as a change in ethanol drinking behavior. Future studies should delve into further ethanol-related behaviors and the mechanism(s) of action of these ethanol-responsive lncRNAs.

Here, we demonstrated that mutating and screening top-ranked ethanol-responsive hub lncRNA genes from chronic ethanol exposed mouse hippocampus led to altered ethanol drinking behavior in all of the generated TAKO cohorts. Among the mutant lines tested, Pitt4 appears to be the ideal target to generate a true breeding line for further studies. This would permit studying additional ethanol-related behaviors as well as an in-depth molecular analysis to discern the potential function(s) and mechanism of action(s) for this novel lncRNA. The data presented here add to the growing body of literature supporting the hypothesis that expression of specific lncRNAs is important for mediating addiction-related behaviors relevant to human health (63, 69–71).

## Data Availability

The datasets presented in this study can be found in online repositories. The names of the repository/repositories and accession number(s) can be found in the article/Supplementary Material.
