# Supplementary figures and images for: Hippocampal ceRNA networks from chronic intermittent ethanol vapor-exposed male mice and functional analysis of top-ranked lncRNA genes for ethanol drinking phenotypes
Source: Adv Drug Alcohol Res. 2022 Dec 5;2:10831. doi: 10.3389/adar.2022.10831 (PMC10004261; doi:10.3389/adar.2022.10831)

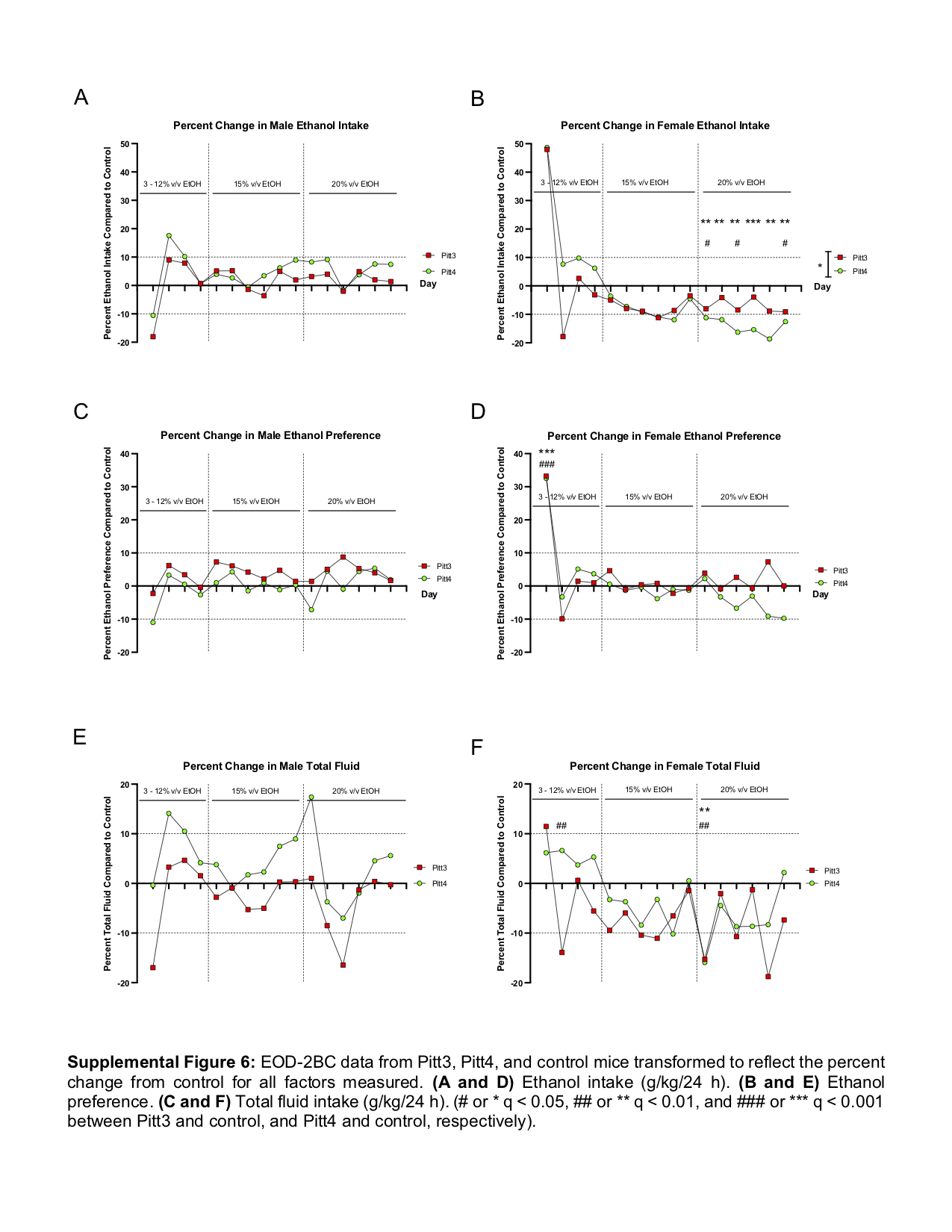

Supplement: Supplementary file 1 [file Image6.jpg]

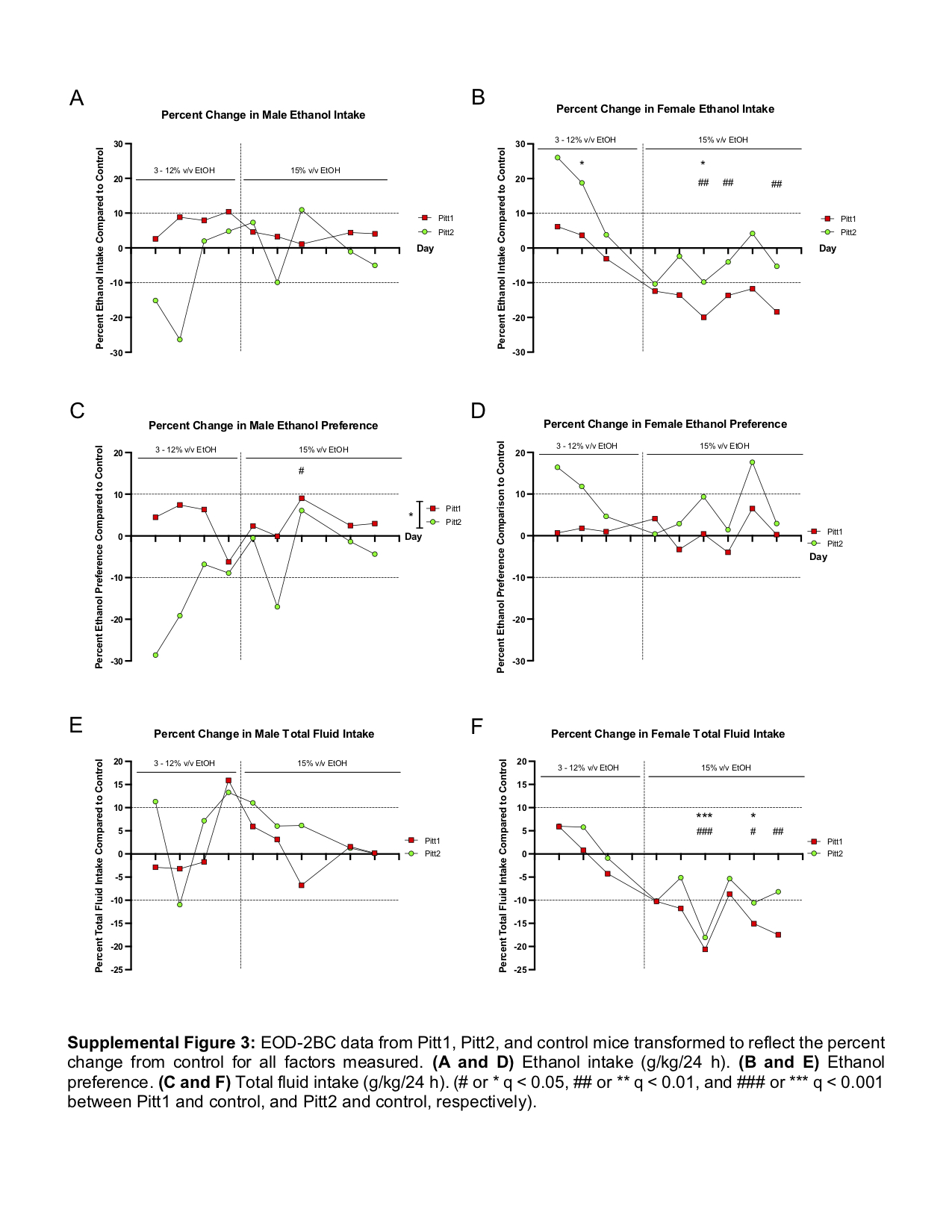

Supplement: Supplementary file 2 [file Image3.jpg]

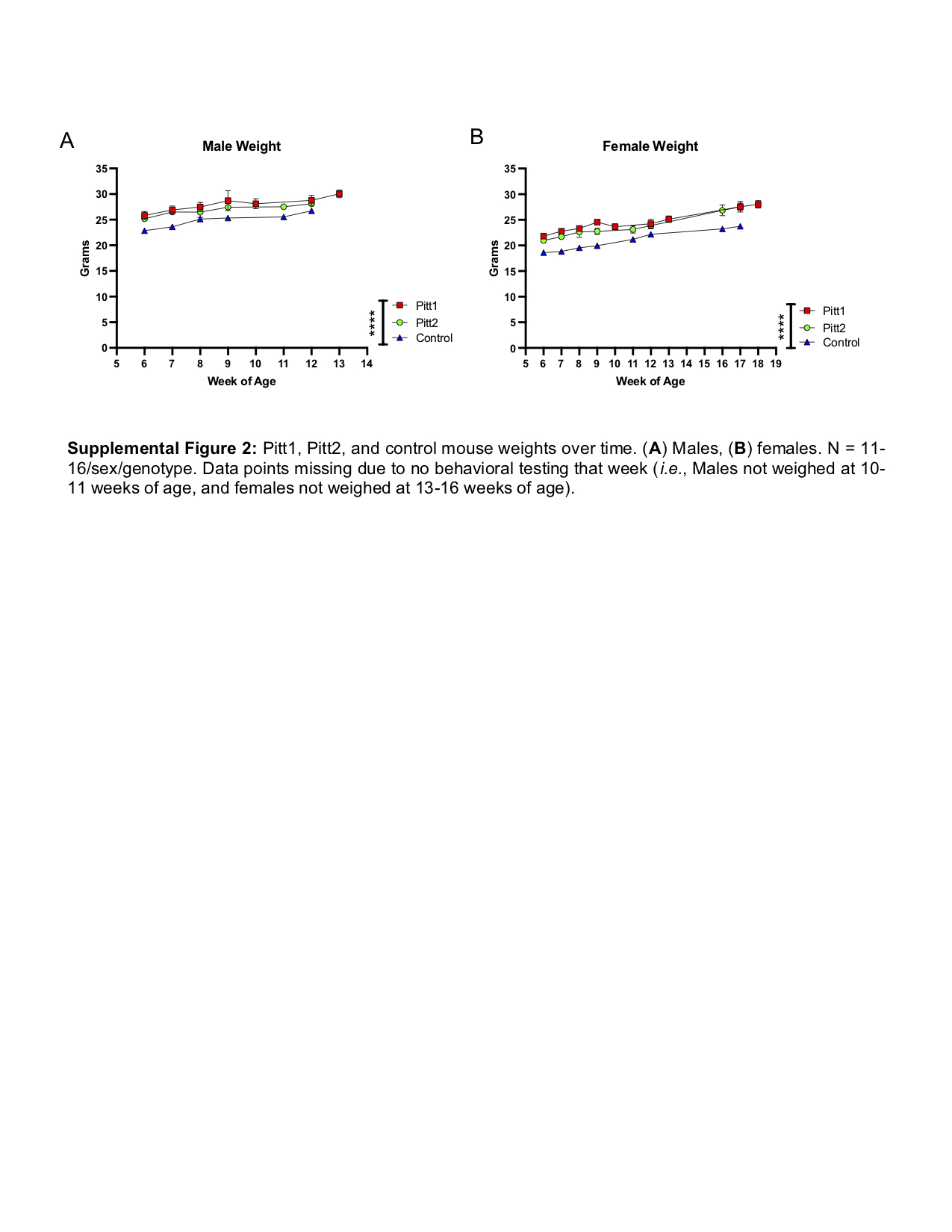

Supplement: Supplementary file 3 [file Image2.jpg]

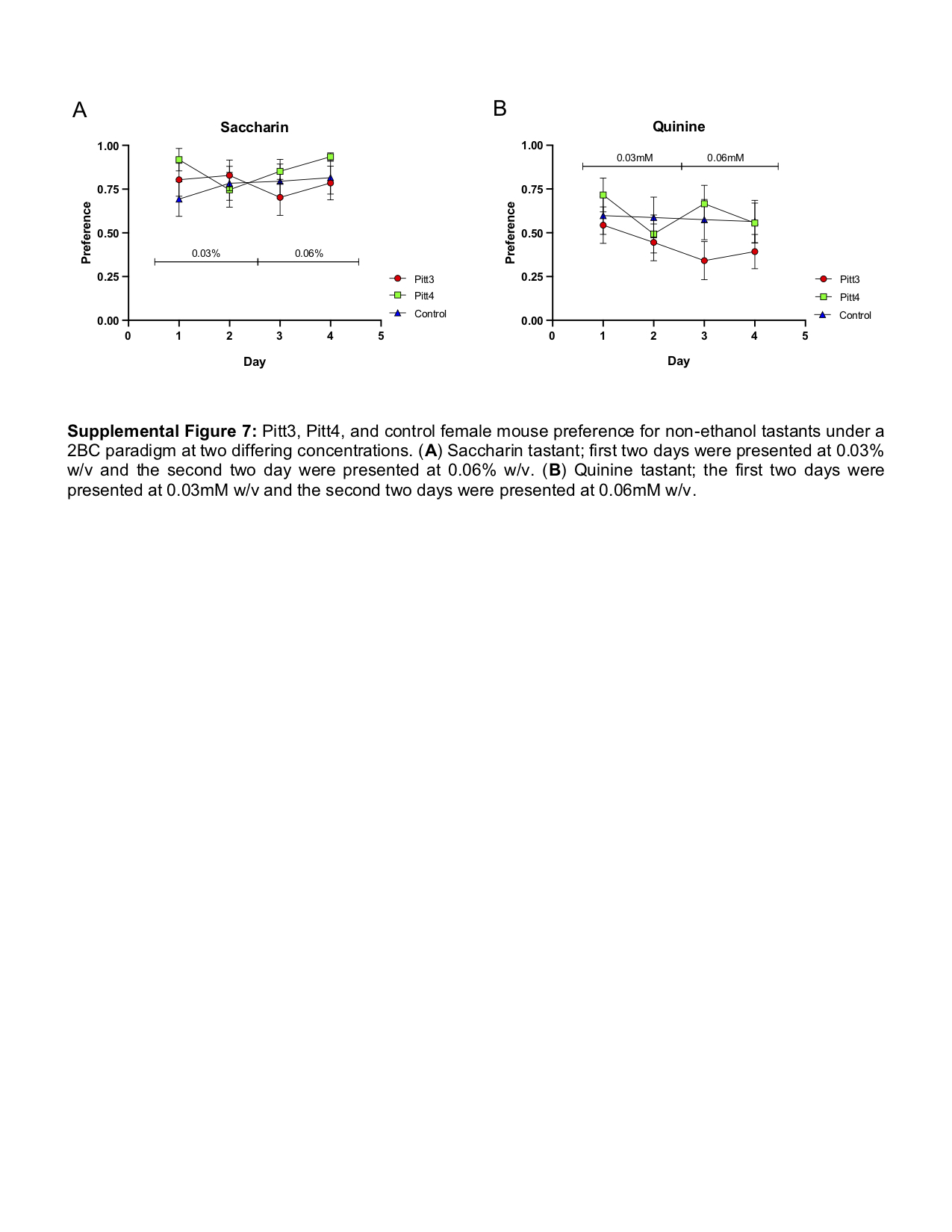

Supplement: Supplementary file 5 [file Image7.jpg]

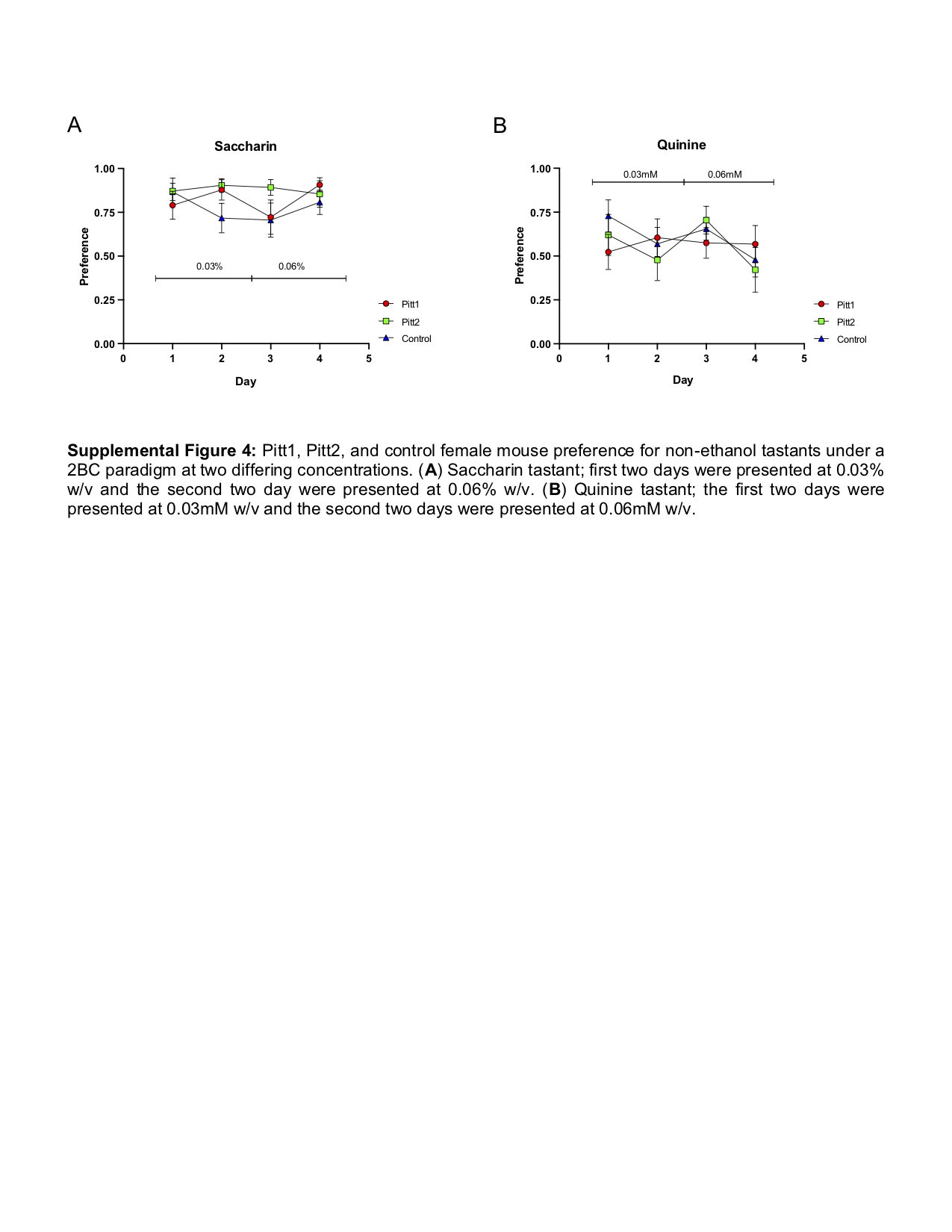

Supplement: Supplementary file 6 [file Image4.jpg]

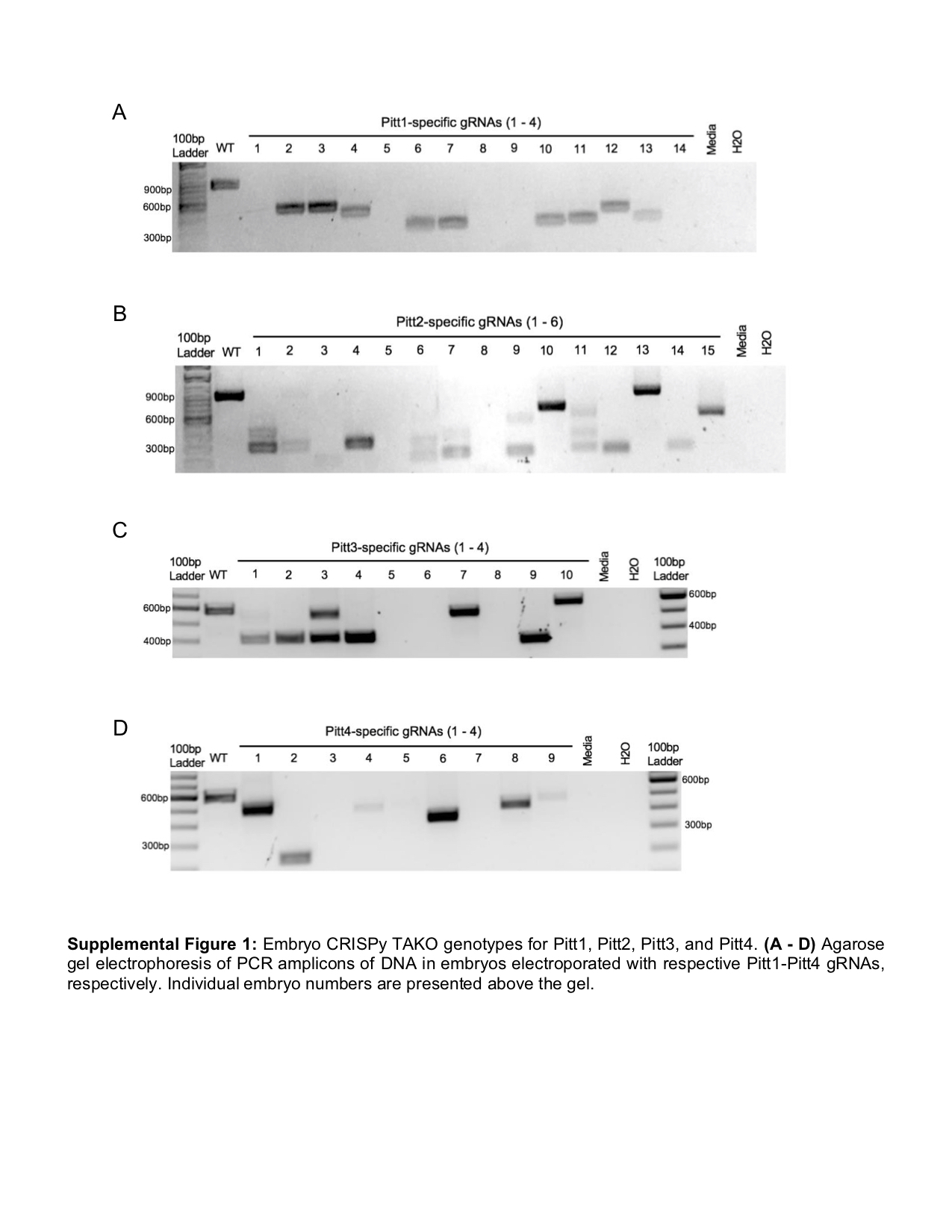

Supplement: Supplementary file 7 [file Image1.jpg]
