# Supplementary material for: Hippocampal ceRNA networks from chronic intermittent ethanol vapor-exposed male mice and functional analysis of top-ranked lncRNA genes for ethanol drinking phenotypes
Source: Adv Drug Alcohol Res. 2022 Dec 5;2:10831. doi: 10.3389/adar.2022.10831 (PMC10004261; doi:10.3389/adar.2022.10831)
Supplement: Supplementary file 4 [file Table1.docx]

**Supplemental Table 1.** gRNA target sites, and PCR and RT-PCR primer sequences. All sequences are written in a 5’ to 3’ orientation. Note: underlined sequence in each gRNA target site is the protospacer adjacent motif.

| **Name** | **Sequence** |
| --- | --- |
| Pitt1 #1 gRNA | CACAACTGGAAGCAAAGACG AGG |
| Pitt1 #2 gRNA | AGATGAGACTCGAGACATCT GGG |
| Pitt1 #3 gRNA | GAACTGTAAACCATTAAACT GGG |
| Pitt1 #4 gRNA | CTTGGAACCAACTCAGTGAG AGG |
| Pitt2 #1 gRNA | AGTAGGCCATGAGGTCACAG AGG |
| Pitt2 #2 gRNA | TGTGATAGGCCAGGGTATCA GGG |
| Pitt2 #3 gRNA | TTGAGAATAGGCTTCCACAG AGG |
| Pitt2 #4 gRNA | GTCCCTAACAAGAAAAACCA AGG |
| Pitt2 #5 gRNA | CCCCTCCACAGGGGGCATGG AGG |
| Pitt2 #6 gRNA | GTAGTCATCATGGAAATATG AGG |
| Pitt3 #1 gRNA | CTGAGCCAATCACTGTGGCT GGG |
| Pitt3 #2 gRNA | GATGACAGAGCGATCTTACG AGG |
| Pitt3 #3 gRNA | TGTGTCCACATCATCGAGTG GGG |
| Pitt3 #4 gRNA | GCAGTTGGTGATTGCTGTGG AGG |
| Pitt4 #1 gRNA | GAACTTCAGTGAAACGTGAG AGG |
| Pitt4 #2 gRNA | GTTGGGTTTTAATTGCGCCA GGG |
| Pitt4 #3 gRNA | ACTTTATGGACAGTATGGGG TGG |
| Pitt4 #4 gRNA | GATCAGCACATGTGTCCGTG TGG |
| Pitt1 F1 PCR primer | ﻿AGCCCATGGAATGCTTGACA |
| Pitt1 R1 PCR primer | TGAGTAATGCTGGCCTT |
| Pitt1 F2 RT-PCR primer | CTGGCTGCTGGTGAAAGAGA |
| Pitt1 R2 RT-PCR primer | GGGAAACTCCAAAGCTTCCG |
| Pitt1 F3 RT-PCR primer | CCAGGTCCTAGATGTTTTGGGG |
| Pitt1 R3 RT-PCR primer | AGAGCAAAATACCATTAGAATAGCAC |
| Pitt2 F1 PCR primer | ﻿CATGTGACTGGTGAAGGCCT |
| Pitt2 R1 PCR primer | AATGAGTCCCAGGAAGTGCG |
| Pitt3 F1 PCR primer | CCATGCACTTCTCAAAGTCAGA |
| Pitt3 R1 PCR primer | TCAATGAGCTCCCCCTTTCC |
| Pitt3 F2 RT-PCR primer | AGATCGCTCTGTCATCCCCT |
| Pitt3 R2 RT-PCR primer | GGCTGCTTTTCTTCATGGCT |
| Pitt3 F3 RT-PCR primer | TGAAGCTCTCCATGACAGGGA |
| Pitt3 R3 RT-PCR primer | ATGAGGTACGTGCAATGCCA |
| Pitt4 F1 PCR primer | AGAGAGGCTGAGACGTGGAT |
| Pitt4 R1 PCR primer | CAACCCTTCCCTGGCATCTT |
| Pitt4 F2 RT-PCR primer | TCCGGAAGTAAGGCCTCTCA |
| Pitt4 R2 RT-PCR primer | TGGCCCAGTGGTTTAAAGCA |
| Pitt4 F3 RT-PCR primer | GCCTCTCACCTTGTTGGCAA |
| Pitt4 R3 RT-PCR primer | GAAAGAAACCGGCACCTCCT |
| *MyD88* F1 RT-PCR primer | GGTGGCCAGAGTGGAAAGCAGTGTCCC |
| *MyD88* R1 RT-PCR primer | GAAACAACCACCACCATGCGGCGACA |
